# *In vitro* interactions of proton pump inhibitors and azoles against pathogenic fungi

**DOI:** 10.3389/fcimb.2024.1296151

**Published:** 2024-01-17

**Authors:** Lujuan Gao, Xuqiong Xia, Xiao Gong, Heng Zhang, Yi Sun

**Affiliations:** ^1^ Department of Dermatology, Zhongshan Hospital (Xiamen), Fudan University, Xiamen, Fujian, China; ^2^ Department of Dermatology, Zhongshan Hospital, Fudan University, Shanghai, China; ^3^ Department of Dermatology, Ninth People’s Hospital Affiliated to Shanghai Jiao Tong University School of Medicine, Shanghai, China; ^4^ Department of Dermatology, Jingzhou Hospital Affiliated to Yangtze University, Jingzhou, Hubei, China

**Keywords:** fungi, *Aspergillus*, *Candida*, azole, dematiaceous fungi, proton pump inhibitors, pantoprazole, omeprazole

## Abstract

**Introduction:**

Azole resistance has been increasingly reported and become an issue for clinical managements of invasive mycoses. New strategy with combination therapy arises as a valuable and promising alternative option. The aim of the present study is to investigate the *in vitro* combinational effect of proton pump inhibitors (PPIs) and azoles against pathogenic fungi.

**Methods:**

*In vitro* interactions of PPIs including omeprazole (OME), lansoprazole (LAN), pantoprazole (PAN), and rabeprazole (RAB), and commonly used azoles including itraconazole (ITC), posaconazole (POS), voriconazole (VRC) and fluconazole (FLC), were investigated via broth microdilution chequerboard procedure adapted from the CLSI M27-A3 and M38-A2. A total of 67 clinically isolated strains, namely 27 strains of *Aspergillus* spp., 16 strains of *Candida* spp., and 24 strains of dematiaceous fungi, were studied. *C. parapsilosis* (ATCC 22019) and *A. flavus* (ATCC 204304) was included to ensure quality control.

**Results:**

PPIs individually did not exert any significant antifungal activity. The combination of OME with ITC, POS, or VRC showed synergism against 77.6%, 86.6%, and 4% strains of tested pathogenic fungi, respectively, while synergism of OME/FLC was observed in 50% strains of *Candida* spp. Synergism between PAN and ITC, POS, or VRC was observed against 47.8%, 77.6% and 1.5% strains of tested fungi, respectively, while synergism of PNA/FLC was observed in 50% strains of *Candida* spp. Synergism of LAN with ITC, POS, or VRC was observed against 86.6%, 86.6%, and 3% of tested strains, respectively, while synergism of LAN/FLC was observed in 31.3% strains of *Candida* spp. Synergy of the combination of RAB with ITC, POS, or VRC was observed against 25.4%, 64.2%, and 4.5% of tested strains, respectively, while synergism of RAB/FLC was observed in 12.5% of *Candida* spp.. Among PPIs, synergism was least observed between RAB and triazoles, while among triazoles, synergism was least observed between VRC and PPIs. Among species, synergy was much more frequently observed in *Aspergillus* spp. and dematiaceous fungi as compared to *Candida* spp. Antagonism between PPIs with ITC or VRC was occasionally observed in *Aspergillus* spp. and dematiaceous fungi. It is notable that PPIs combined with azoles showed synergy against azole resistant *A. fumigatus*, and resulted in category change of susceptibility of ITC and POS against *Candida* spp.

**Discussion:**

The results suggested that PPIs combined with azoles has the potential to enhance the susceptibilities of azoles against multiple pathogenic fungi and could be a promising strategy to overcome azole resistance issues. However, further investigations are warranted to study the combinational efficacy in more isolates and more species, to investigate the underlying mechanism of interaction and to evaluate the potential for concomitant use of these agents in human.

## 1 Introduction

Invasive fungal infections have emerged as a major clinical challenge and imposed economic burden to the health care system worldwide due to the widespread application of immunosuppressive and immunomodulation-based therapies (Enoch et al., 2017). Invasive candidiasis and aspergillosis are the most common invasive yeast and mold infection, respectively(Erjavec et al., 2009; Brown et al., 2012; Gonzalez-Lara and Ostrosky-Zeichner, 2020). *Candida albicans* and *Aspergillus fumigatus* are always the most frequent organisms isolated. However, a rise of non-albicans *Candida* and non-fumigatus *Aspergillus* with reduced susceptibility to common available antifungal agents was noted, which constitutes a substantial proportion of invasive candidiasis and invasive aspergillosis (Lamoth et al., 2015; Gonzalez-Lara and Ostrosky-Zeichner, 2020). The mortality rates of invasive candidiasis and invasive aspergillosis were reported to be as high as 75% (Brown et al., 2012; Kullberg and Arendrup, 2015) and 95% (Brown et al., 2012), respectively. In addition, opportunistic infection by dematiaceous fungi is also being increasingly recognized and reported (Zeng et al., 2007; Li et al., 2011; Patel et al., 2013). Triazoles are still the main choice for invasive fungal infections(Campoy and Adrio, 2017). However, azole resistance has been increasingly reported (Prasad et al., 2017; Hadrich and Ayadi, 2018). New strategy with combination therapy arises as a valuable and promising alternative option due to its potential to increase the efficacy of current antifungals and to reduce the probability of resistance.

Cellular pH homeostasis plays a critical role in fungal cell physiology. Abundant fungal plasma membrane and organellar pumps are responsible for intracellular pH regulation, maintenance of ionic balance and electrochemical proton gradients, which are essential for nutrient uptake, cell growth and virulence(Monk et al., 1995b). The proton pump inhibitors exert antifungal effect and may potentially reverse the acquired resistance to azoles (Afeltra and Verweij, 2003; Monk et al., 2005). Hence, targeting proton pumps might be a promising target for broad spectrum combinational strategy with conventional antifungals. The FDA-approved proton pump inhibitors (PPIs) such as omeprazole (OME), lansoprazole (LAN), pantoprazole (PAN), and rabeprazole (RAB) are widely applied for the treatment of digestive disorders such as peptic ulcer disease and gastrointestinal hemorrhage(Asim Syed and Abbas Naqvi, 2016). Previous studies have demonstrated that OME exerted fungicidal growth inhibition against *Saccharomyces cerevisiae* and *Candida albicans* (Monk et al., 1995a), and that activated LAN showed hyphal growth inhibition of *C. albicans* (Biswas et al., 2001). In addition, PPIs have been demonstrated to act synergistically with fluconazole (FLC) against *C. albicans in vitro* and *in vivo* (Lu et al., 2020). However, the combinational effects of PPI and azoles against other species of *Candida* spp., dematiaceous fungi and *Aspergillus* spp. were still unknown. Therefore, it is tempting to investigate the combined effect of FDA-approved PPIs and triazoles against other pathogenic fungi. In the present study, the *in vitro* interactions between PPIs and commonly used itraconazole (ITC), posaconazole (POS), voriconazole (VRC) and FLC against pathogenic fungi were investigated.

## 2 Materials and methods

### 2.1 Fungal strains, antifungals and chemical agents

A total of 67 clinically isolated strains, namely 27 strains of *Aspergillus* spp. (14 strains of *A. fumigatus* including four azole-resistant *A. fumigatus* strains harboring the association of a tandem repeat sequence and punctual mutation of the Cyp51A gene (TR34/L98H and TR46/Y121F/T289A, 12 strains of *A. flavus*, and 1 strain of *A. niger*), 16 strains of *Candida* spp. (9 strains of *C. albicans*, 2 strains of *C. krusei*, 2 strains of *C. parasilosis*, 3 strains of *C. tropicalis)*, 18 strains of *E. dermatitidis*, 3 strains of *Fonsecaea. monophora*, 2 strains of *Phialophora americana* and 1 strain of *Phialophora macrospora* were studied*. C. parapsilosis* (ATCC 22019) and *A. flavus* (ATCC 204304) was included to ensure quality control. All fungal strains were identified by microscopic morphology and by molecular sequencing of the internal transcribed spacer (ITS) ribosomal DNA (rDNA) (Glass and Donaldson, 1995). For identification of *Aspergillus* spp., additional molecular sequence of β-tubulin and calmodulin was required (Hong et al., 2005; Samson and Varga, 2009).

The experimental agents including OME (CAS: 73590-58-6), PAN (CAS: 102625-70-7), LAN (CAS: 103577-45-3), RAB (CAS: 117976-89-3) and ITC (CAS: 84625-61-6) were purchased in powder form from MedChemExpress (MCE), NJ, USA; VRC (CAS: 137234-62-9) was obtained from Solarbio, Beijing, China; POS (CAS: 171228-49-2) was obtained from Meilunbio, Dalian, China; and FLC (CAS: 86386-73-4) was obtained from Selleck Chemicals, Houston, TX, USA. The powder was dissolved diluted in dimethyl sulfoxide as stock solutions (3200 μg/ml).

### 2.2 *In vitro* interactions of PPIs and azoles against pathogenic fungi

The broth microdilution chequerboard procedure based on the CLSI M27-A3 (Clinical and laboratory standards institute, 2008), M38-A2 (Clinical and laboratory standards institute, 2017) standard and previously published protocols (Gao et al., 2020) was performed. Conidia were harvested from fungal cultures grown on Sabouraud dextrose agar (SDA) for 2, 3, and 5 days for *Candida* spp., *Aspergillus* spp., and dematiaceous fungi, respectively. Subsequently, the conidia were resuspended in sterile distilled water containing 0.03% Triton and diluted to the concentration of 1-5×10^6^ spores/ml and 2-5×10^6^ spores/ml for yeast and filamentous fungi, respectively. The suspension was than diluted 1000 times with RPMI-1640 to achieve a two-fold suspension more concentrated than the density needed or to approximately 2-4×10^3^ spores/ml (Clinical and laboratory standards institute, 2008) for yeast and 1-3×10^4^ spores/ml (Clinical and laboratory standards institute, 2017) for filamentous fungi, respectively. The working concentration ranges of PPIs were 2-128 μg/ml for all tested species. The working concentration ranges of tested azoles against both *Aspergillus* spp. and dematiaceous fungi were 0.06-8μg/ml for ITC and VRC, and 0.03-4μg/ml for POS, respectively, except that for azole resistant *A. fumigatus*, the working concentration of all azoles were adjusted to 0.5-32μg/ml. The working concentration ranges of tested azoles against *Candida* spp. were 0.06-16μg/ml for ITC and VRC, 0.03-8μg/ml for POS, and 0.25-64μg/ml for FLC, respectively.

As described, a 50 μl of serial diluted PPIs was inoculated horizontally and another 50 μl of serial diluted azoles was inoculated vertically in the 96-well plate, which contained 100μl prepared inoculum suspension. Interpretation of results was performed after incubation at 35°C for 24h for *Candida* spp., 48h for, *Aspergillus* spp., and 72h for dematiaceous fungi, respectively. The MICs applied for the evaluation against *Candida* spp. were determined as the lowest concentration resulting in 50% inhibition of growth (Clinical and laboratory standards institute, 2008). The MICs applied for the evaluation against filamentous fungi were determined as the lowest concentration resulting in 100% inhibition of growth (Clinical and laboratory standards institute, 2017). The combination interaction between PPIs and azoles was classified according to the fractional inhibitory concentration index (FICI), which was calculated by the formula: FICI=(Ac/Aa)+(Bc/Ba), where Ac and Bc are the MICs of antifungal drugs in combination, and Aa and Ba are the MICs of antifungal drugs A and B alone (Tobudic et al., 2010). An FICI of ≤0.5 indicates synergy, an FICI of >0.5 to ≤4 indicates no interaction (indifference), and an FICI of >4 indicates antagonism (Odds, 2003). All tests were performed in triplicate.

## 3 Results

### 3.1 *In vitro* interactions between OME and azoles against pathogenic fungi

**Figure 1:**
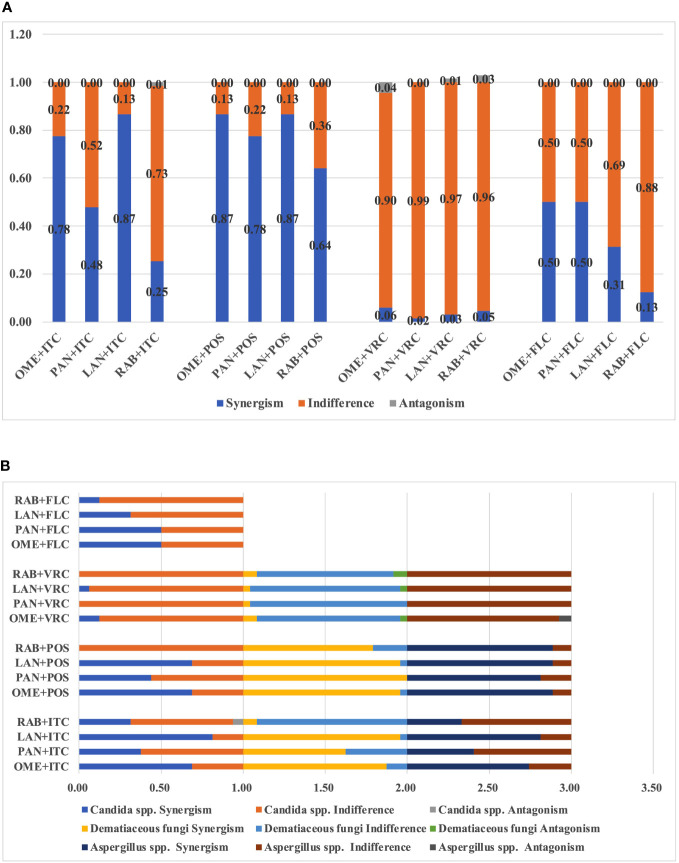
**(A)** Interaction profile between PPIs and azoles against all tested fungi. **(B)** Interaction profile between PPIs and azoles among different species.

The median and range of MICs of OME and azoles alone or in combination were listed in **Table 1**. OME alone exhibited limited antifungal effect against all tested species. However, the median MICs of azoles and OME in the combination revealed up to eight-fold and 128-fold reduction compared to that when tested alone, respectively. The summarized interaction profile between OME and azoles was shown in **Figures 1A, B**. Synergy of OME/ITC was observed in 20 strains of *Aspergillus* spp., 21 strains of dematiaceous fungi and 11 strains of *Candida* spp., respectively. Synergy of OME/POS was shown in 24 strains of *Aspergillus* spp., 23 strains of dematiaceous fungi and 11 strains of *Candida* spp., respectively. In contrast, synergy between OME and VRC was only observed in 2 strains of dematiaceous fungi and 2 strains of *Candida* spp. Synergy of OME/FLC was observed in 8 strains of *Candida* spp. Antagonism was only observed in the combination of OME/VRC against 3 strains of tested fungi. In total, the combination of OME/ITC and OME/POS showed synergism against 77.6% and 86.6% strains of pathogenic fungi, respectively, while synergism of OME/FLC was observed in 50% strains of *Candida* spp. However, synergism was rarely observed in the combination of OME/VRC.

**Table 1:** Summary of drug interaction for the combination of OME and azoles.

	Median MIC^a^ (range) of durg (*μ*g/mL)					FICI ^b^results (n)		
	Alone			In combination		S	I	A
	Azoles	OME		Azoles	OME			
*A. fumigatus* (n=14)								
ITC	2 (1->32)	>128	0.5 (0.13->32)		32 (2->128)	10	4	0
POS	1 (0.5-2)	>128	0.25 (0.06-1)		32 (2-128)	11	3	0
VRC	0.5 (0.25-32)	>128	1.25 (0.25-32)		2	0	13	1
Non-fumigatus *Aspergillus* (n=13)								
ITC	1 (0.5-2)	>128	0.25 (0.06-1)		32 (2-128))	10	3	0
POS	1 (0.5-1)	>128	0.125 (0.06-0.25)		32 (16-64)	13	0	0
VRC	0.5 (0.25-2)	>128	0.5 (0.25-4)		2	0	12	1
Dematiaceous fungi (n=24)								
ITC	1 (0.13-2)	>128 (64->128)	0.13 (0.03-0.5)		16 (16-64)	21	3	0
POS	0.5 (0.25-1)	>128 (64->128)	0.06 (0.03-0.25)		16 (8-64)	23	1	0
VRC	0.19 (0.06-2)	>128 (64->128)	0.19 (0.06-0.5)		2 (2-128)	2	21	1
*Candida* spp. (n=16)								
ITC	1 (0.13-2)	≥128	0.38 (0.13-1)		32 (2-64)	11	5	0
POS	0.5 (0.13-2)	≥128	0.13 (0.06-2)		32 (2-64)	11	5	0
VRC	0.25 (0.13-4)	≥128	0.13 (0.13-4)		2 (2-64)	2	14	0
FLC	4 (0.25-32)	≥128	1 (0.25-16)		2 (1-8)	8	8	0

a The MIC is the concentration achieving 100% growth inhibition for *Aspergillus* spp. and dematiaceous fungi and 50% growth inhibition for *Candida* spp., respectively.

b FICI, fractional inhibitory concentration index; S, synergy (FICI of ≤ 0.5); I, no interaction (indifference) (0.5<FICI ≤ 4); A, antagonism (FICI of >4).

### 3.2 *In vitro* interactions between PAN and azoles against pathogenic fungi

The median and range of MICs of PAN and azoles alone or in combination were listed in **Table 2**. PAN alone exhibited limited antifungal effect against all tested species. However, the median MICs of azoles and PAN in the combination revealed up to eight-fold and 128-fold reduction compared to that when tested alone, respectively. The summarized interaction profile between PAN and azoles was shown in **Figures 1A, B**. Synergy of PAN/ITC was observed in 11 strains of *Aspergillus* spp., 15 strains of dematiaceous fungi and six strains of *Candida* spp., respectively. The synergy of PAN/POS was shown in 22 strains of *Aspergillus* spp., 23 strains of dematiaceous fungi and 7 strains of *Candida* spp., respectively. In contrast, synergy between PAN and VRC was only observed in 1 strains of dematiaceous fungi. Synergy of PAN/FLC was observed in 8 strains of *Candida* spp. No antagonism was observed in the combination of PAN with azoles. In total, PAN synergized with ITC, POS, VRC and FLC against 47.8%, 77.6%, 1.5% and 50% of tested strains, respectively.

**Table 2:** Summary of drug interaction for the combination of PAN and azoles.

	Median MIC^a^ (range) of durg (*μ*g/mL)				FICI ^b^results (n)		
	Alone		In combination		S	I	A
	Azoles	PAN	Azoles	PAN			
*A. fumigatus* (n=14)							
ITC	1 (0.5->32)	>128	0.5 (0.25->32)	64 (64->128)	5	9	0
POS	1 (0.5-2)	>128	0.125 (0.06-2)	64 (2-128)	10	4	0
VRC	0.5 (0.25-32)	>128	0.5 (0.5-32)	2	0	14	0
Non-fumigatus *Aspergillus* (n=13)							
ITC	0.5 (0.5-1)	>128	0.125 (0.06-0.5)	64 (16-128))	6	7	0
POS	1 (0.5-1)	>128	0.25 (0.06-0.25)	64 (16-64)	12	1	0
VRC	0.5 (0.25-2)	>128	0.5 (0.5-2)	2	0	13	0
Dematiaceous fungi (n=24)							
ITC	1 (0.13-2)	>128 (64->128)	0.25 (0.03-2)	32 (2-64)	15	9	0
POS	0.5 (0.25-1)	>128 (64->128)	0.13 (0.03-0.25)	32 (16-64)	23	1	0
VRC	0.19 (0.06-2)	>128 (64->128)	0.25 (0.06-1)	2 (2->128)	1	23	0
*Candida* spp. (n=16)							
ITC	1 (0.13-2)	>128	0.5 (0.06-1)	2 (2-128)	**6**	10	0
POS	0.5 (0.13-2)	>128	0.19 (0.06-2)	32 (2-64)	7	9	0
VRC	0.25 (0.13-4)	>128	0.25 (0.13-4)	2	0	16	0
FLC	4 (0.25-32)	>128	1 (0.25-16)	2 (1-16)	8	8	0

a The MIC is the concentration achieving 100% growth inhibition for *Aspergillus* spp. and dematiaceous fungi and 50% growth inhibition for *Candida* spp., respectively.

b FICI, fractional inhibitory concentration index; S, synergy (FICI of ≤ 0.5); I, no interaction (indifference) (0.5<FICI ≤ 4); A, antagonism (FICI of >4).

### 3.3 *In vitro* interactions between LAN and azoles against pathogenic fungi

The median and range of MICs of LAN and azoles alone or in combination were listed in **Table 3**. LAN alone exhibited limited antifungal effect against all tested species. However, the median MICs of azoles and LAN in the combination revealed up to 11-fold and 128-fold reduction compared to that when tested alone, respectively. The summarized interaction profile between LAN and azoles was shown in **Figures 1A, B**. Synergy of LAN/ITC was observed in 22 strains of *Aspergillus* spp., 23 strains of dematiaceous fungi and 13 strains of *Candida* spp., respectively. The synergy of LAN/POS was shown in 24 strains of *Aspergillus* spp., 23 strains of dematiaceous fungi and 11 strains of *Candida* spp., respectively. In contrast, synergy between LAN and VRC was only observed in one strains of dematiaceous fungi and one strains of *Candida* spp. Synergy of LAN/FLC was observed in five strains of *Candida* spp. Antagonism of LAN/VRC was observed in one strains of dematiaceous fungi. In total, synergy of the combination of LAN with ITC and POS was observed against 86.6% of tested strains, while synergy of LAN/VRC and LAN/FLC was only observed in 3% and 31.3% tested strains, respectively.

**Table 3:** Summary of drug interaction for the combination of LAN and azoles.

	Median MIC^a^ (range) of durg (*μ*g/mL)				FICI^b^ results (n)		
	Alone		In combination		S	I	A
	Azoles	LAN	Azoles	LAN			
*A. fumigatus* (n=14)							
ITC	1 (0.5->32)	>128	0.25 (0.13->32)	32 (2->128)	10	4	0
POS	1 (0.5-2)	>128	0.09 (0.06-1)	16 (8-32)	11	3	0
VRC	0.5 (0.25-32)	>128	0.5 (0.25-32)	2	0	14	0
Non-fumigatus *Aspergillus* (n=13)							
ITC	0.5 (0.25-1)	>128	0.06 (0.06-0.25)	32 (16-128))	12	1	0
POS	1 (0.5-1)	>128	0.13 (0.06-0.25)	16 (8-32)	13	0	0
VRC	0.5 (0.5-2)	>128	0.5 (0.5-4)	2	0	13	0
Dematiaceous fungi (n=24)							
ITC	1 (0.13-2)	>128 (64->128)	0.13 (0.03-0.5)	24 (16-64)	23	1	0
POS	0.5 (0.25-1)	>128 (64->128)	0.06 (0.03-0.5)	16 (8-32)	23	1	0
VRC	0.19 (0.06-2)	>128 (64->128)	0.25 (0.13-1)	2 (2->128)	1	22	1
*Candida* spp. (n=16)							
ITC	1 (0.13-2)	>128	0.25 (0.06-1)	24 (2-64)	13	3	0
POS	0.5 (0.13-2)	>128	0.13 (0.06-2)	16 (2-32)	11	5	0
VRC	0.25 (0.13-4)	>128	0.13 (0.13-4)	2 (1-64)	1	15	0
FLC	4 (0.25-32)	>128	1.5 (0.25-16)	2 (0.5-8)	5	11	0

a The MIC is the concentration achieving 100% growth inhibition for *Aspergillus* spp. and dematiaceous fungi and 50% growth inhibition for *Candida* spp., respectively.

b FICI, fractional inhibitory concentration index; S, synergy (FICI of ≤ 0.5); I, no interaction (indifference) (0.5<FICI ≤ 4); A, antagonism (FICI of >4).

### 3.4 *In vitro* interactions between RAB and azoles against pathogenic fungi

The median and range of MICs of RAB and azoles alone or in combination were listed in **Table 4**. RAB alone exhibited limited antifungal effect against all tested species. However, the median MICs of azoles and RAB in the combination revealed up to 7.7-fold and 128-fold reduction compared to that when tested alone, respectively. The summarized interaction profile between RAE and azoles was shown in **Figures 1A, B**. Synergy of RAB/ITC was observed in 10 strains of *Aspergillus* spp., two strains of dematiaceous fungi and five strains of *Candida* spp., respectively. The synergy of RAB/POS was shown in 24 strains of *Aspergillus* spp., and 19 strains of dematiaceous fungi. Synergy between RAB and VRC was only observed in 1 strains of *Aspergillus* spp., and two strains of dematiaceous fungi. Synergy of RAB/FLC was observed in two strains of *Candida* spp. Antagonism of RAB/VRC was observed in two strains of dematiaceous fungi and antagonism of RAB/ITC was observed in one strain of *Candida* spp. In total, synergy of the combination of RAB with ITC, POS, VRC or FLC was observed against 25.4%, 64.2%, 4.5% and 12.5% of tested strains, respectively.

**Table 4:** Summary of drug interaction for the combination of RAB and azoles.

	Median MIC^a^ (range) of durg (*μ*g/mL)				FICI^b^ results (n)		
	Alone		In combination		S	I	A
	Azoles	RAB	Azoles	RAB			
A. fumigatus (n=14)							
ITC	2 (1->32)	>128	0.75 (0.25->32)	64 (2->128)	6	8	0
POS	1 (0.5-2)	>128	0.25 (0.06-2)	32 (2-64)	12	2	0
VRC	0.75 (0.25-32)	>128	0.75 (0.25-32)	2	0	14	0
Non-fumigatus *Aspergillus* (n=13)							
ITC	1 (0.5-4)	>128	1 (0.25-2)	2 (2-128))	4	9	0
POS	1 (0.5-1)	>128	0.13 (0.06-1)	32 (2-64)	12	1	0
VRC	0.5 (0.25-2)	>128	0.5 (0.25-2)	2 (2-32)	1	12	0
Dematiaceous fungi (n=24)							
ITC	1 (0.13-2)	>128	0.5 (0.13-1)	2 (2->128)	2	22	0
POS	0.5 (0.25-1)	>128	0.13 (0.03-0.5)	32 (4->128)	19	5	0
VRC	0.19 (0.06-2)	>128	0.25 (0.06-1)	2 (2-32)	2	20	2
*Candida* spp. (n=16)							
ITC	1 (0.13-2)	>128	0.5 (0.13-2)	2 (2-64)	5	10	1
POS	0.5 (0.13-2)	>128	0.5 (0.13-2)	2 (2-32)	0	16	0
VRC	0.25 (0.13-4)	>128	0.25 (0.13-4)	2	0	16	0
FLC	4 (0.25-32)	>128	3 (0.25-16)	3 (1-4)	2	14	0

a The MIC is the concentration achieving 100% growth inhibition for *Aspergillus* spp. and dematiaceous fungi and 50% growth inhibition for *Candida* spp., respectively.

b FICI, fractional inhibitory concentration index; S, synergy (FICI of ≤ 0.5); I, no interaction (indifference) (0.5<FICI ≤ 4); A, antagonism (FICI of >4).

## 4 Discussion

As the present study revealed, among species, synergy was much more frequently observed in *Aspergillus* spp. and dematiaceous fungi as compared to *Candida* spp. (**Tables S1-S15**; **Figure 1B**). However, it's notable that PPIs combined with azoles resulted in category change of ITC, VRC and FLC susceptibilities in *Candida* spp. (**Tables S1-S5**) and that ITC category change was most often observed in the LAN/ITC combination, while FLC category change in *Candida* spp. was most commonly observed in the PAN/FLC combination. In addition, the combination with PPIs and POS or ITC exhibited synergism against two azole-resistant *A. fumigatus* strains that harboring the association of a tandem repeat sequence and punctual mutation of the Cyp51A gene (TR34/L98H and TR46/Y121F/T289A), resulting in up to 8-fold reduction in MICs of azoles (**Tables S6-9**).

Although, clinically, PPIs combined with FLC have been successfully applied for the treatment of *candida* esophagitis combined acute oesophageal necrosis (Pereira et al., 2013; Chen et al., 2016), paradoxical interactions between PPIs and FLC have been reported against the yeast *Candida*. OME, RAB, PAN and esomeprazole have also been shown to antagonize the growth inhibition effect of FLC (Kaneko et al., 2013; Urai et al., 2014; Liu and Kohler, 2016). However, recently PPIs, including OME, RAB, PAN, esomeprazole and ilaprazole, have been demonstrated to act synergistically with FLC against *C. albicans* both *in vitro* and *in vivo* (Lu et al., 2020). The present study also demonstrated variable interaction profiles between PPIs and triazole against multiple species of *Candida, Aspergillus* and dematiaceous fungi. Among PPIs, synergism was least observed between RAB and triazoles, while among triazoles, synergism was least observed between VRC and PPIs (**Table 5**; **Figure 1**). In addition, antagonism was most commonly observed between VRC and PPIs (**Table 5**; **Figure 1**).

**Table 5:** Summary of drug interaction for the combination of PPIs and azoles.

PPIs	ITC (n=67)			POS (n=67)			VRC (n=67)			FLC (n=16)		
	S	I	A	S	I	A	S	I	A	S	I	A
OME	52 (77.6%)	15 (22.4%)	0	58 (86.6%)	9 (13.4%)	0	4 (6%)	60 (89.6%)	3	8 (50%)	8 (50%)	0
PAN	32 (47.8%)	35 (52.2%)	0	52 (77.6%)	15 (22.4%)	0	1 (1.5%)	66 (98.5%)	0	8 (50%)	8 (50%)	0
LAN	58 (86.6%)	9 (13.4%)	0	58 (86.6%)	9 (13.4%)	0	2 (3%)	64 (97%)	1	5 (31.3%)	11 (68.7%)	0
RAB	17 (25.4%)	49 (73.1%)	1	43 (64.2%)	24 (35.8%)	0	3 (4.5%)	62 (95.5%)	2	2 (12.5%)	14 (87.5%)	0

Triazoles targets ergosterol biosynthetic enzyme and results in diminished ergosterol in fungal plasma membrane. This disruption in membrane function corresponds to fungal growth inhibition(Peyton et al., 2015). However, upregulated expression of multidrug efflux pumps correlates with decreased susceptibility of azoles(Fraczek et al., 2013). The plasma membrane structure is crucial for the proper localization and function of efflux pumps. It has been demonstrated that both ergosterol and sphingolipid are crucial for maintenance of plasma membrane structure (Urbanek et al., 2022). Inhibition of sphingolipid synthesis results in significantly elevated susceptibility to azoles and diminished efflux pump levels (Gao et al., 2018; Urbanek et al., 2022). The FDA-approved proton pump inhibitors (PPIs) have been shown to have an inhibitory effect against fatty acid synthases (FAS) and inhibited proliferation of pancreatic cancer cells (Fako et al., 2015). In all live creatures, FAS are central to metabolism since that fatty acids and their biosynthesis are essential for the survival, representing building blocks of lipids membranes and also energy reserves of a cell and precursors to second messenger molecules(Schweizer and Hofmann, 2004; White et al., 2005). Specifically, in fungi, fatty acids are building blocks for sphingolipid (Lomakin et al., 2007). The inhibitory effect of PPIs on FAS might provide one mechanistic basis for the synergy between azoles and PPIs.

It has been shown that PPIs target and inhibit plasma membrane ATPase, including fungal plasma membrane ATPase Pam1p(Monk et al., 1995a). Studies have demonstrated that targeting plasma Pam1p increases the potencies of azole drugs and circumvents azole resistance (Monk et al., 2005). In addition, Pma1p inhibition may affect pathways downstream of Vacuolar-ATPase(V-ATPases) function (Hayek et al., 2014), which is responsible for acidifying and maintaining the pH of intracellular compartments and is important for fundamental cellular processes such as mTOR, Notch, and Wnt signaling(Vasanthakumar and Rubinstein, 2020). Previous research demonstrated that V-ATPase is required for antifungal resistance and virulence of *Candida glabrata*, and inhibition of V-ATPase exerts a synergistic effect with azole antifungal agents (Minematsu et al., 2019). Although PPIs are primarily applied as irreversible blockers of the plasma membrane ATPase, they have also been demonstrated to be effective on V-ATPases at higher concentrations (Mattsson et al., 1991; Moriyama et al., 1993). In addition, ergosterol also plays a regulatory role in fungal V-ATPase function(Zhang et al., 2010). Therefore, the effect of PPIs on both plasma and vacuolar ATPase might provide another plausible mechanism for synergism of PPIs and azoles.

However, antagonism between PPIs and azoles have also been reported (Liu and Kohler, 2016) and observed in our study. Previous study has shown that OME induces CDR1 expression and disturbs the anti-*Candida* activity of FLC(Urai et al., 2014). In our study, no antagonism was observed between PPIs and FLC against tested *Candida* strains. These demonstrated the complex combinatorial effect of PPIs and azoles could be dependent on the tested strains as well as the specific type of azoles used. In addition, in the present study, synergy between PPIs and POS or ITC was more frequently observed than that observed in the combinations of PPIs and VRC or FLC (**Table 5**, **Figure 1**). The underlying mechanism may lies in the fact that VRC is a synthetic derivative of FLC by the substitution of a triazole group with a fluoropyrimidine moiety and by the addition of a methyl group to the propyl backbone (Sabo and Abdel-Rahman, 2000), while POS is structurally an analogue of itraconazole with a 1,3-dioxolone backbone (Kauffman et al., 2007), which might explain the discrepancy of the interaction profile of PPIs with VRC as compared to ITC or POS.

As mentioned above, synergism was least observed in RAB-triazoles combinations and VRC-PPIs combinations, while antagonism was most commonly observed in VRC-PPIs combinations (**Figure 1**). Antagonism was not observed between POS or FLC and PPIs. These provides preliminary information for therapeutic decision by clinicians when patients received both PPIs and triazoles treatment. For patients under triazoles treatment, RAB is the least priority to be chose among PPIs for gastric disease. For patients under VRC treatment, other category instead of PPIs may offer a better option. However, further investigations are warranted to study the combinational efficacy in more isolates and more species, to investigate the underlying mechanism of interaction and to evaluate the potential for concomitant use of these agents in human.

In summary, the present study investigated the combinational efficacies of PPIs and triazoles against multiple pathogenic fungi. PPIs exerted favorable synergistic effect with triazoles, although antagonism was occasionally observed in several strains. Additionally, PPIs have the potential to reverse azoles resistance of pathogenic fungi, which provided new promising strategies to overcome the resistance issue. However, combinational effect of PPIs with triazoles may be dependent on the tested strains as well as the specific type of azoles used.

## Data availability statement

The original contributions presented in the study are included in the article/**Supplementary Material**. Further inquiries can be directed to the corresponding author.

## Author contributions

LG: Writing – original draft, Conceptualization, Funding acquisition. XX: Writing – original draft, Investigation, Formal analysis. XG: Methodology, Writing – original draft. HZ: Investigation, Writing – original draft, Methodology. YS: Writing – review & editing, Funding acquisition, Supervision.

## References

[B1] Afeltra J. Verweij P. E. (2003). Antifungal activity of nonantifungal drugs. Eur. J. Clin. Microbiol. Infect. Dis. 22, 397–407. doi: 10.1007/s10096-003-0947-x 12884072

[B2] Asim Syed I. A. Abbas Naqvi S. H. (2016). Proton pump inhibitors use; beware of side-effects. J. Pak. Med. Assoc. 66, 1314–1318. 27686311

[B3] Biswas S. K. Yokoyama K. Kamei K. Nishimura K. Miyaji M. (2001). Inhibition of hyphal growth of *Candida albicans* by activated lansoprazole, a novel benzimidazole proton pump inhibitor. Med. Mycol. 39, 283–285. doi: 10.1080/mmy.39.3.283.285 11446532

[B4] Brown G. D. Denning D. W. Gow N. A. Levitz S. M. Netea M. G. White T. C. (2012). Hidden killers: human fungal infections. Sci. Transl. Med. 4, 165rv113. doi: 10.1126/scitranslmed.3004404 23253612

[B5] Campoy S. Adrio J. L. (2017). Antifungals. Biochem. Pharmacol. 133, 86–96. doi: 10.1016/j.bcp.2016.11.019 27884742

[B6] Chen K. H. Weng M. T. Chou Y. H. Lu Y. F. Hsieh C. H. (2016). Epigastric distress caused by esophageal candidiasis in 2 patients who received sorafenib plus radiotherapy for hepatocellular carcinoma: case report. Med. (Baltimore). 95, e3133. doi: 10.1097/MD.0000000000003133 PMC4839949 26986168

[B7] Clinical and laboratory standards institute (2008). Reference method for broth dilution antifungal susceptibility testing of yeasts; approved standard. 3rd ed (Wayne, PA: CLSI).

[B8] Clinical and laboratory standards institute (2017). Reference method for broth dilution antifungal susceptibility testing of filamentous fungi. 3rd ed (Wayne, PA: Clinical and Laboratory Standards Institute).

[B9] Enoch D. A. Yang H. Aliyu S. H. Micallef C. (2017). The changing epidemiology of invasive fungal infections. Methods Mol. Biol. 1508, 17–65. doi: 10.1007/978-1-4939-6515-1_2 27837497

[B10] Erjavec Z. Kluin-Nelemans H. Verweij P. E. (2009). Trends in invasive fungal infections, with emphasis on invasive aspergillosis. Clin. Microbiol. Infect. 15, 625–633. doi: 10.1111/j.1469-0691.2009.02929.x 19673973

[B11] Fako V. E. Wu X. Pflug B. Liu J. Y. Zhang J. T. (2015). Repositioning proton pump inhibitors as anticancer drugs by targeting the thioesterase domain of human fatty acid synthase. J. Med. Chem. 58, 778–784. doi: 10.1021/jm501543u 25513712 PMC4306520

[B12] Fraczek M. G. Bromley M. Buied A. Moore C. B. Rajendran R. Rautemaa R.. (2013). The cdr1B efflux transporter is associated with non-cyp51a-mediated itraconazole resistance in *Aspergillus fumigatus* . J. Antimicrob. Chemother. 68, 1486–1496. doi: 10.1093/jac/dkt075 23580559

[B13] Gao J. Wang H. Li Z. Wong A. H. Wang Y. Z. Guo Y.. (2018). *Candida albicans* gains azole resistance by altering sphingolipid composition. Nat. Commun. 9, 4495. doi: 10.1038/s41467-018-06944-1 30374049 PMC6206040

[B14] Gao L. Sun Y. Yuan M. Li M. Zeng T. (2020). *In vitro* and *in vivo* study on the synergistic effect of minocycline and azoles against pathogenic fungi. Antimicrob. Agents Chemother. 64, e00290–20. doi: 10.1128/AAC.00290-20 32253207 PMC7269466

[B15] Glass N. L. Donaldson G. C. (1995). Development of primer sets designed for use with the PCR to amplify conserved genes from filamentous ascomycetes. Appl. Environ. Microbiol. 61, 1323–1330. doi: 10.1128/aem.61.4.1323-1330.1995 7747954 PMC167388

[B16] Gonzalez-Lara M. F. Ostrosky-Zeichner L. (2020). Invasive candidiasis. Semin. Respir. Crit. Care Med. 41, 3–12. doi: 10.1055/s-0040-1701215 32000280

[B17] Hadrich I. Ayadi A. (2018). Epidemiology of antifungal susceptibility: Review of literature. J. Mycol. Med. 28, 574–584. doi: 10.1016/j.mycmed.2018.04.011 29773435

[B18] Hayek S. R. Lee S. A. Parra K. J. (2014). Advances in targeting the vacuolar proton-translocating ATPase (V-ATPase) for anti-fungal therapy. Front. Pharmacol. 5, 4. doi: 10.3389/fphar.2014.00004 24478704 PMC3902353

[B19] Hong S. B. Go S. J. Shin H. D. Frisvad J. C. Samson R. A. (2005). Polyphasic taxonomy of *Aspergillus fumigatus* and related species. Mycologia 97, 1316–1329. doi: 10.1080/15572536.2006.11832738 16722222

[B20] Kaneko Y. Fukazawa H. Ohno H. Miyazaki Y. (2013). Combinatory effect of fluconazole and FDA-approved drugs against *Candida albicans* . J. Infect. Chemother. 19, 1141–1145. doi: 10.1007/s10156-013-0639-0 23807392

[B21] Kauffman C. A. Malani A. N. Easley C. Kirkpatrick P. (2007). Posaconazole. Nat. Rev. Drug Discovery 6, 183–184. doi: 10.1038/nrd2270 17396290

[B22] Kullberg B. J. Arendrup M. C. (2015). Invasive candidiasis. N. Engl. J. Med. 373, 1445–1456. doi: 10.1056/NEJMra1315399 26444731

[B23] Lamoth F. Juvvadi P. R. Steinbach W. J. (2015). Histone deacetylase inhibition as an alternative strategy against invasive aspergillosis. Front. Microbiol. 6, 96. doi: 10.3389/fmicb.2015.00096 25762988 PMC4329796

[B24] Li D. M. Li R. Y. De Hoog G. S. Sudhadham M. Wang D. L. (2011). Fatal *Exophiala* infections in China, with a report of seven cases. Mycoses 54, e136–e142. doi: 10.1111/j.1439-0507.2010.01859.x 20337943

[B25] Liu N. N. Kohler J. R. (2016). Antagonism of Fluconazole and a Proton Pump Inhibitor against *Candida albicans* . Antimicrob. Agents Chemother. 60, 1145–1147. doi: 10.1128/AAC.02043-15 26596946 PMC4750695

[B26] Lomakin I. B. Xiong Y. Steitz T. A. (2007). The crystal structure of yeast fatty acid synthase, a cellular machine with eight active sites working together. Cell 129, 319–332. doi: 10.1016/j.cell.2007.03.013 17448991

[B27] Lu M. Yan H. Yu C. Yuan L. Sun S. (2020). Proton pump inhibitors act synergistically with fluconazole against resistant *Candida albicans* . Sci. Rep. 10, 498. doi: 10.1038/s41598-019-57174-4 31949170 PMC6965112

[B28] Mattsson J. P. Vaananen K. Wallmark B. Lorentzon P. (1991). Omeprazole and bafilomycin, two proton pump inhibitors: differentiation of their effects on gastric, kidney and bone H(+)-translocating ATPases. Biochim. Biophys. Acta 1065, 261–268. doi: 10.1016/0005-2736(91)90238-4 1647821

[B29] Minematsu A. Miyazaki T. Shimamura S. Nishikawa H. Nakayama H. Takazono T.. (2019). Vacuolar proton-translocating ATPase is required for antifungal resistance and virulence of *Candida glabrata* . PloS One 14, e0210883. doi: 10.1371/journal.pone.0210883 30673768 PMC6343876

[B30] Monk B. C. Mason A. B. Abramochkin G. Haber J. E. Seto-Young D. Perlin D. S. (1995a). The yeast plasma membrane proton pumping ATPase is a viable antifungal target. I. Effects of the cysteine-modifying reagent omeprazole. Biochim. Biophys. Acta 1239, 81–90. doi: 10.1016/0005-2736(95)00133-N 7548148

[B31] Monk B. C. Mason A. B. Kardos T. B. Perlin D. S. (1995b). Targeting the fungal plasma membrane proton pump. Acta Biochim. Pol. 42, 481–496. doi: 10.18388/abp.1995_4901 8852338

[B32] Monk B. C. Niimi K. Lin S. Knight A. Kardos T. B. Cannon R. D.. (2005). Surface-active fungicidal D-peptide inhibitors of the plasma membrane proton pump that block azole resistance. Antimicrob. Agents Chemother. 49, 57–70. doi: 10.1128/AAC.49.1.57-70.2005 15616276 PMC538910

[B33] Moriyama Y. Patel V. Ueda I. Futai M. (1993). Evidence for a common binding site for omeprazole and N-ethylmaleimide in subunit A of chromaffin granule vacuolar-type H(+)-ATPase. Biochem. Biophys. Res. Commun. 196, 699–706. doi: 10.1006/bbrc.1993.2306 8240346

[B34] Odds F. C. (2003). Synergy, antagonism, and what the chequerboard puts between them. J. Antimicrob. Chemother. 52, 1. doi: 10.1093/jac/dkg301 12805255

[B35] Patel A. K. Patel K. K. Darji P. Singh R. Shivaprakash M. R. Chakrabarti A. (2013). *Exophiala dermatitidis* endocarditis on native aortic valve in a postrenal transplant patient and review of literature on *E. dermatitidis* infections. Mycoses 56, 365–372. doi: 10.1111/myc.12009 23013169

[B36] Pereira O. Figueira-Coelho J. Picado B. Costa J. N. (2013). Black oesophagus. BMJ Case Rep. 2013. doi: 10.1136/bcr-2012-008188 PMC3603927 23365174

[B37] Peyton L. R. Gallagher S. Hashemzadeh M. (2015). Triazole antifungals: a review. Drugs Today (Barc). 51, 705–718. doi: 10.1358/dot.2015.51.12.2421058 26798851

[B38] Prasad R. Banerjee A. Shah A. H. (2017). Resistance to antifungal therapies. Essays. Biochem. 61, 157–166. doi: 10.1042/EBC20160067 28258238

[B39] Sabo J. A. Abdel-Rahman S. M. (2000). Voriconazole: a new triazole antifungal. Ann. Pharmacother. 34, 1032–1043. doi: 10.1345/aph.19237 10981251

[B40] Samson R. A. Varga J. (2009). What is a species in *aspergillus* ? Med. Mycol. 47 Suppl 1, S13–S20. doi: 10.1080/13693780802354011 19255907

[B41] Schweizer E. Hofmann J. (2004). Microbial type I fatty acid synthases (FAS): major players in a network of cellular FAS systems. Microbiol. Mol. Biol. Rev. 68, 501–517. doi: 10.1128/MMBR.68.3.501-517.2004 15353567 PMC515254

[B42] Tobudic S. Kratzer C. Lassnigg A. Graninger W. Presterl E. (2010). *In vitro* activity of antifungal combinations against *Candida* albicans biofilms. J. Antimicrob. Chemother. 65, 271–274. doi: 10.1093/jac/dkp429 19996142

[B43] Urai M. Kaneko Y. Niki M. Inoue M. Tanabe K. Umeyama T.. (2014). Potent drugs that attenuate anti-*Candida albicans* activity of fluconazole and their possible mechanisms of action. J. Infect. Chemother. 20, 612–615. doi: 10.1016/j.jiac.2014.06.004 25009090

[B44] Urbanek A. K. Muraszko J. Derkacz D. Lukaszewicz M. Bernat P. Krasowska A. (2022). The role of ergosterol and sphingolipids in the localization and activity of *candida albicans*' Multidrug transporter cdr1p and plasma membrane ATPase pma1p. Int. J. Mol. Sci. 23, 9975. doi: 10.3390/ijms23179975 36077373 PMC9456455

[B45] Vasanthakumar T. Rubinstein J. L. (2020). Structure and roles of V-type ATPases. Trends Biochem. Sci. 45, 295–307. doi: 10.1016/j.tibs.2019.12.007 32001091

[B46] White S. W. Zheng J. Zhang Y. M. (2005). The structural biology of type II fatty acid biosynthesis. Annu. Rev. Biochem. 74, 791–831. doi: 10.1146/annurev.biochem.74.082803.133524 15952903

[B47] Zeng J. S. Sutton D. A. Fothergill A. W. Rinaldi M. G. Harrak M. J. De Hoog G. S. (2007). Spectrum of clinically relevant *Exophiala* species in the United States. J. Clin. Microbiol. 45, 3713–3720. doi: 10.1128/JCM.02012-06 17596364 PMC2168524

[B48] Zhang Y. Q. Gamarra S. Garcia-Effron G. Park S. Perlin D. S. Rao R. (2010). Requirement for ergosterol in V-ATPase function underlies antifungal activity of azole drugs. PloS Pathog. 6, e1000939. doi: 10.1371/journal.ppat.1000939 20532216 PMC2880581

