# Supplementary material for: In vitro interactions of proton pump inhibitors and azoles against pathogenic fungi
Source: Front Cell Infect Microbiol. 2024 Jan 17;14:1296151. doi: 10.3389/fcimb.2024.1296151 (PMC10831725; doi:10.3389/fcimb.2024.1296151)
Supplement: Supplementary file 1 [file Table_1.docx]

TABLE S1 MICs and FICIs results with the combinations of omeprazole and azoles against *Candida* spp.

|  | MIC^a^ (mg/mL) for | | | | | | | | |  |
| --- | --- | --- | --- | --- | --- | --- | --- | --- | --- | --- |
|  | Agent alone | | | | | Combinations^b^ (µg/mL) | | | |  |
| Strains | OME | ITC | VOR | POS | FLC | OME/ITC | OME/VOR | OME/POS | OME/FLC | |
| *C. albicans* |  |  |  |  |  |  |  |  |  | |
| CA1 | >128 | 0.5 | 0.125 | 0.5 | 32 | 32/0.5(1.13,I) | 2/0.125(1.01,I) | 32/0.125(0.38,S) | 4/4(0.14,S)^c^ | |
| CA2 | >128 | 2 | 0.25 | 0.125 | 4 | 32/0.5(0.38,S)^c^ | 2/0.25(1.01,I) | 2/0.0625(0.51,I) | 1/1(0.25,S)^d^ | |
| CA3 | >128 | 0.5 | 0.5 | 0.5 | 0.5 | 32/0.125(0.38,S)^d^ | 2/0.125(0.26,S)^f^ | 64/0.125(<0.50,S) | 4/0.5(1.02,I) | |
| CA4 | >128 | 0.125 | 0.125 | 0.125 | 0.25 | 2/0.125(1.01,I) | 2/0.125(1.01,I) | 32/0.0625(0.63,I) | 1/0.25(1.00,I) | |
| CA5 | >128 | 1 | 0.25 | 2 | 8 | 2/1(1.01,I) | 2/0.25(1.01,I) | 64/0.5(<0.50,S) | 8/8(1.03,I) | |
| CA6 | >128 | 0.5 | 0.125 | 0.5 | 32 | 64/0.125(<0.50,S)^d^ | 2/0.125(1.01,I) | 32/0.125(0.38,S) | 4/2(0.08,S)^e^ | |
| CA7 | >128 | 0.5 | 0.125 | 2 | 1 | 8/0.125(0.28,S)^d^ | 2/0.125(1.01,I) | 2/2(1.01,I) | 2/1(1.01,I) | |
| CA8 | >128 | 1 | 1 | 0.5 | 2 | 8/0.5(0.53,I)^c^ | 2/1(1.01,I) | 32/0.125(0.38,S) | 2/0.5(0.26,S) | |
| CA9 | >128 | 0.5 | 0.5 | 0.5 | 2 | 2/0.5(1.01,I) | 2/0.5(1.01,I) | 2/0.5(1.01,I) | 1/1(>0.50,I) | |
| *C. krusei* |  |  |  |  |  |  |  |  |  | |
| ATCC6258 | >128 | 1 | 0.25 | 0.5 | 32 | 32/0.25(0.38,S) | 2/0.25(1.01,I) | 64/0.0625(0.38,S) | 4/16(0.52,I) | |
| CK1 | >128 | 2 | 0.5 | 1 | 16 | 32/0.5(0.38,S) | 64/0.125(<0.50,S) | 32/0.125(0.25,S) | 8/4(0.28,S) | |
| *C. parapsilosis* | | | | | | | | | |  |
| ATCC22019 | >128 | 1 | 0.125 | 0.25 | 2 | 64/0.125(0.38,S) | 2/0.125(1.01,I) | 8/0.0625(0.28,S) | 4/2(1.02,I) | |
| CP1 | 128 | 2 | 0.125 | 0.5 | 16 | 32/0.5(0.50S) | 2/0.125(1.02,I) | 32/0.125(0.50,S) | 1/4(0.26,S)^c^ | |
| *C. tropicalis* |  |  |  |  |  |  |  |  |  | |
| CT1 | >128 | 1 | 4 | 0.5 | 4 | 64/0.25(<0.50,S) | 2/4(1.01,I) | 32/0.125(0.38,S) | 1/1(0.25,S)^d^ | |
| CT2 | >128 | 0.5 | 2 | 0.5 | 4 | 64/0.125(<0.50,S) | 2/2(1.01,I) | 64/0.125(<0.50,S) | 2/1(0.26,S)^d^ | |
| CT3 | >128 | 2 | 0.125 | 0.5 | 2 | 32/0.5(0.38,S) | 2/0.125(1.01,I) | 2/0.5(1.01,I) | 1/1(>0.50,I) | |

^a^ The MIC is the concentration achieving 50% growth inhibition;

^b^ FICI results are shown in parentheses. S, synergy (FICI of ≤ 0.5); I, no interaction (indifference) (0.5<FICI≤4); A, antagonism (FICI of >4). For FICI calculations, the concentration of 256μg/ml were used when MICs were >128μg/ml.

^c^ Category change from resistance to susceptible dose dependent (SDD) of ITC and FLC

^d^ Category change from SDD to susceptible of ITC and FLC

^e^ Category change from resistance to susceptible of ITC and FLC

^f^ Category change from intermediate to susceptible of VRC

^g^ Category change from resistance to intermediate of VRC

Susceptible/SDD/resistant is defined as an MIC ≤2/4/≥8 mg/L of fluconazole for *C. albicans*, *C. tropicalis* and *C. parapsilosis*, respectively (1).
Susceptible/intermidiate/resistant is defined as an MIC ≤0.125/0.25-0.5/≥1 mg/L of voriconazole for *C. albicans, C. tropicalis* and *C. parapsilosis*, and an MIC ≤0.5/1/≥2 mg/L of voriconazole for *C. krusei* (1).
Susceptible/SDD/resistant is defined as an MIC ≤0.125/0.25 – 0.5/≥1 mg/L of itraconazole for *C. albicans*(1).

Category change was analyzed for those species with CLSI clinical breakpoint.

TABLE S2 MICs and FICIs results with the combinations of pantoprazole and azoles against *Candida* spp.

|  | MIC^a^ (mg/mL) for | | | | | | | | | |
| --- | --- | --- | --- | --- | --- | --- | --- | --- | --- | --- |
|  | Agent alone | | | | | | Combinations^b^ (µg/mL) | | | |
| Strains | PAN | ITC | VOR | POS | FLC | PAN/ITC | | PAN/VOR | PAN/POS | PAN/FLC |
| *C. albicans* |  |  |  |  |  |  | |  |  |  |
| CA1 | >128 | 0.5 | 0.125 | 0.5 | 32 | 2/0.5(1.01,I) | | 2/0.125(1.01,I) | 64/0.125(<0.50,S) | 16/4(0.19,S)^c^ |
| CA2 | >128 | 2 | 0.25 | 0.125 | 4 | 2/1(0.51,I) | | 2/0.25(1.01,I) | 2/0.125(1.01,I) | 1/0.5(0.13,S)^d^ |
| CA3 | >128 | 0.5 | 0.5 | 0.5 | 0.5 | 2/0.5(101,I) | | 2/0.5(1.01,I) | 32/0.5(1.13,I) | 1/0.5(1.00,I) |
| CA4 | >128 | 0.125 | 0.125 | 0.125 | 0.25 | 2/0.0625(0.51,I) | | 2/0.125(1.01,I) | 2/0.0625(0.51,I) | 2/0.25(1.01,I) |
| CA5 | >128 | 1 | 0.25 | 2 | 8 | 2/1(1.01,I) | | 2/0.25(1.01,I) | 32/0.5(0.38,S) | 1/4(>0.50,I)^c^ |
| CA6 | >128 | 0.5 | 0.125 | 0.5 | 32 | 2/1(2.01,I) | | 2/0.125(1.01,I) | 32/0.125(0.38,S) | 4/2(0.08,S)^e^ |
| CA7 | >128 | 0.5 | 0.125 | 2 | 1 | 32/0.125(0.38,S)^d^ | | 2/0.125(1.01,I) | 2/2(1.01,I) | 4/1(1.02,I) |
| CA8 | >128 | 1 | 1 | 0.5 | 2 | 4/0.125(0.14,S)^e^ | | 2/1(1.01,I) | 16/0.25(0.56,I) | 2/1(0.51,I) |
| CA9 | >128 | 0.5 | 0.5 | 0.5 | 2 | 2/0.5(1.01,I) | | 2/0.25(0.51,I) | 2/0.5(1.01,I) | 2/0.5(0.26,S) |
| *C. krusei* |  |  |  |  |  |  | |  |  |  |
| ATCC6258 | >128 | 1 | 0.25 | 0.5 | 32 | 64/0.25(<0.50,S) | | 2/0.25(1.01,I) | 64/0.125(<0.50,S) | 2/16(0.51,I) |
| CK | >128 | 2 | 0.5 | 1 | 16 | 64/0.25(0.38,S) | | 2/0.25(0.51,I) | 32/0.5(0.63,I) | 2/4(0.26,S) |
| *C. parapsilosis* | | | | | | | | | | |
| ATCC22019 | >128 | 1 | 0.125 | 0.25 | 2 | 64/0.25(<0.50,S) | | 2/0.125(1.01,I) | 32/0.0625(0.38,S) | 16/2(1.06,I) |
| CP | >128 | 2 | 0.125 | 0.5 | 16 | 64/0.5(<0.50,S) | | 2/0.125(1.01,I) | 32/0.125(0.38,S) | 1/4(0.25,S)^c^ |
| *C. tropicalis* |  |  |  |  |  |  | |  |  |  |
| CT1 | >128 | 1 | 4 | 0.5 | 4 | 128/0.5(1.00,I） | | 2/4(1.01,I) | 64/0.125(<0.50,S) | 2/0.25(0.07,S)^d^ |
| CT2 | >128 | 0.5 | 2 | 0.5 | 4 | 2/0.5(1.01,I) | | 2/2(1.01,I) | 64/0.25(0.75,I) | 1/0.25(0.07,S)^d^ |
| CT3 | >128 | 2 | 0.125 | 0.5 | 2 | 2/1(0.51,I) | | 2/0.125(1.01,I) | 2/0.5(1.01,I) | 1/1(0.504,I) |

^a^ The MIC is the concentration achieving 50% growth inhibition;

^b^ FICI results are shown in parentheses. S, synergy (FICI of ≤ 0.5); I, no interaction (indifference) (0.5<FICI≤4); A, antagonism (FICI of >4). For FICI calculations, the concentration of 256μg/ml were used when MICs were >128μg/ml.

^c^ Category change from resistance to susceptible dose dependent (SDD) of ITC and FLC

^d^ Category change from SDD to susceptible of ITC and FLC

^e^ Category change from resistance to susceptible of ITC and FLC

^f^ Category change from intermediate to susceptible of VRC

^g^ Category change from resistance to intermediate of VRC

Susceptible/SDD/resistant is defined as an MIC ≤2/4/≥8 mg/L of fluconazole for *C. albicans*, *C. tropicalis* and *C. parapsilosis*, respectively (1).
Susceptible/intermidiate/resistant is defined as an MIC ≤0.125/0.25-0.5/≥1 mg/L of voriconazole for *C. albicans, C. tropicalis* and *C. parapsilosis*, and an MIC ≤0.5/1/≥2 mg/L of voriconazole for *C. krusei* (1).
Susceptible/SDD/resistant is defined as an MIC ≤0.125/0.25 – 0.5/≥1 mg/L of itraconazole for *C. albicans*(1).

Category change was analyzed for those species with CLSI clinical breakpoint.

TABLE S3 MICs and FICIs results with the combinations of lansoprazole and azoles against *Candida* spp.

|  | MIC^a^ (mg/mL) for | | | | | | | | | |  |
| --- | --- | --- | --- | --- | --- | --- | --- | --- | --- | --- | --- |
|  | Agent alone | | | | | | Combinations^b^ (µg/mL) | | | |  |
| Strains | LAN | ITC | VOR | POS | FLC | LAN/ITC | | LAN/VOR | LAN/POS | LAN/FLC | |
| *C. albicans* |  |  |  |  |  |  | |  |  |  | |
| CA1 | >128 | 0.5 | 0.125 | 0.5 | 32 | 32/0.125(0.38,S)^d^ | | 2/0.125(1.01,I) | 8/0.125(0.28,S) | 4/16(0.52,I) | |
| CA2 | >128 | 2 | 0.25 | 0.125 | 4 | 16/0.125(0.13,S)^e^ | | 2/0.125(0.51,I)^f^ | 2/0.0625(0.51,I) | 2/0.25(0.07,S)^d^ | |
| CA3 | >128 | 0.5 | 0.5 | 0.5 | 0.5 | 8/0.125(0.28,S)^d^ | | 1/0.25(>0.50,I) | 16/0.0625(0.19,S) | 4/0.5(1.02,I) | |
| CA4 | >128 | 0.125 | 0.125 | 0.125 | 0.25 | 2/0.0625(0.51,I) | | 2/0.125(1.01,I) | 2/0.0625(0.51,I) | 0.5/0.25(1,I) | |
| CA5 | >128 | 1 | 0.25 | 2 | 8 | 32/0.25(0.38,S)^c^ | | 2/0.25(1.01,I) | 8/0.25(0.16,S) | 8/8(1.03,I) | |
| CA6 | >128 | 0.5 | 0.125 | 0.5 | 32 | 16/0.0625(0.13,S)^d^ | | 2/0.125(1.01,I) | 16/0.0625(0.19,S) | 8/16(0.53,I) | |
| CA7 | >128 | 0.5 | 0.125 | 2 | 1 | 16/0.125(0.31,S)^d^ | | 2/0.125(1.01,I) | 2/2(1.01,I) | 1/1(1.00,I) | |
| CA8 | >128 | 1 | 1 | 0.5 | 2 | 16/0.25(0.31,S)^c^ | | 2/0.5(0.51,I)^g^ | 16/0.5(1.06,I) | 2/0.5(0.26,S) | |
| CA9 | >128 | 0.5 | 0.5 | 0.5 | 2 | 2/0.5(1.01,I) | | 16/0.125(0.31,S)^f^ | 32/0.125(0.38,S) | 2/0.5(0.26,S) | |
| *C. krusei* |  |  |  |  |  |  | |  |  |  | |
| ATCC6258 | >128 | 1 | 0.25 | 0.5 | 32 | 32/0.25(0.38,S) | | 64/0.125(0.75,I) | 16/0.0625(0.19,S) | 4/16(0.52,I) | |
| CK | >128 | 2 | 0.5 | 1 | 16 | 16/0.5(0.31,S) | | 64/0.25(0.75,I) | 16/0.125(0.19,S) | 2/4(0.26,S) | |
| *C. parapsilosis* | | | | | | | | | | | |
| ATCC22019 | >128 | 1 | 0.125 | 0.25 | 2 | 32/0.25(0.38,S) | | 2/0.125(1.01,I) | 16/0.0625(0.31,S) | 0.5/1(>0.50,I) | |
| CP | >128 | 2 | 0.125 | 0.5 | 16 | 32/0.5(0.38,S) | | 2/0.125(1.01,I) | 4/0.125(0.27,S) | 1/4(0.25,S)^c^ | |
| *C. tropicalis* |  |  |  |  |  |  | |  |  |  | |
| CT1 | >128 | 1 | 4 | 0.5 | 4 | 32/0.25(0.38,S) | | 2/4(1.01,I) | 16/0.125(0.31,S) | 2/4(1.01,I) | |
| CT2 | >128 | 0.5 | 2 | 0.5 | 4 | 64/0.125(<0.50,S) | | 2/2(1.01,I) | 32/0.125(0.38,S) | 1/2(0.504,I)^d^ | |
| CT3 | >128 | 2 | 0.125 | 0.5 | 2 | 32/1(0.63,I) | | 2/0.125(1.01,I) | 2/0.25(0.51,I) | 1/1(0.504,I) | |

^a^ The MIC is the concentration achieving 50% growth inhibition;

^b^ FICI results are shown in parentheses. S, synergy (FICI of ≤ 0.5); I, no interaction (indifference) (0.5<FICI≤4); A, antagonism (FICI of >4);. For FICI calculations, the concentration of 256μg/ml were used when MICs were >128μg/ml.

^c^ Category change from resistance to susceptible dose dependent (SDD) of ITC and FLC

^d^ Category change from SDD to susceptible of ITC and FLC

^e^ Category change from resistance to susceptible of ITC and FLC

^f^ Category change from intermediate to susceptible of VRC

^g^ Category change from resistance to intermediate of VRC

Susceptible/SDD/resistant is defined as an MIC ≤2/4/≥8 mg/L of fluconazole for *C. albicans*, *C. tropicalis* and *C. parapsilosis*, respectively (1).
Susceptible/intermidiate/resistant is defined as an MIC ≤0.125/0.25-0.5/≥1 mg/L of voriconazole for *C. albicans, C. tropicalis* and *C. parapsilosis*, and an MIC ≤0.5/1/≥2 mg/L of voriconazole for *C. krusei* (1).
Susceptible/SDD/resistant is defined as an MIC ≤0.125/0.25 – 0.5/≥1 mg/L of itraconazole for *C. albicans*(1).

Category change was analyzed for those species with CLSI clinical breakpoint.

TABLE S4 MICs and FICIs results with the combinations of rabeprazole and azoles against *Candida* spp.

|  | MIC^a^ (mg/mL) for | | | | | | | | |
| --- | --- | --- | --- | --- | --- | --- | --- | --- | --- |
|  | Agent alone | | | | | Combinations^b^ (µg/mL) | | | |
| Strains | RAB | ITC | VOR | POS | FLC | RAB/ITC | RAB/VOR | RAB/POS | RAB/FLC |
| *C. albicans* |  |  |  |  |  |  |  |  |  |
| CA1 | >128 | 0.5 | 0.125 | 0.5 | 32 | 2/0.5(1.01,I) | 2/0.125(1.01,I) | 2/1(2.01,I) | 2/16(0.51,I) |
| CA2 | >128 | 2 | 0.25 | 0.125 | 4 | 64/0.5(<0.50,S)^c^ | 2/0.25(1.01,I) | 2/0.125(1.01,I) | 2/2(0.51,I)^d^ |
| CA3 | >128 | 0.5 | 0.5 | 0.5 | 0.5 | 2/0.5(1.01,I) | 2/0.25(0.51,I) | 16/0.5(1.06,I) | 4/0.5(1.02,I) |
| CA4 | >128 | 0.125 | 0.125 | 0.125 | 0.25 | 2/0.125(1.01,I) | 2/0.125(1.01,I) | 2/0.125(1.01,I) | 1/0.25(1.00,I) |
| CA5 | >128 | 1 | 0.25 | 2 | 8 | 2/2(2.01,I) | 2/0.25(1.01,I) | 2/1(0.51,I) | 4/8(1.02,I) |
| CA6 | >128 | 0.5 | 0.125 | 0.5 | 32 | 2/0.5(1.01,I) | 2/0.25(2.01,I) | 2/0.5(1.01,I) | 4/16(0.52,I) |
| CA7 | >128 | 0.5 | 0.125 | 2 | 1 | 2/2(4.01,A) | 2/0.125(1.01,I) | 2/2(1.01,I) | 4/1(1.02,I) |
| CA8 | >128 | 1 | 1 | 0.5 | 2 | 64/0.125(0.38,S)^e^ | 2/1(1.01,I) | 2/0.5(1.01,I) | 4/1(0.52,I) |
| CA9 | >128 | 0.5 | 0.5 | 0.5 | 2 | 2/0.5(1.01,I) | 2/0.5(1.01,I) | 32/0.5(1.13,I) | 4/1(0.52,I) |
| *C. krusei* |  |  |  |  |  |  |  |  |  |
| ATCC6258 | >128 | 1 | 0.25 | 0.5 | 32 | 64/0.25(<0.50,S) | 2/0.25(1.01,I) | 2/0.5(1.01,I) | 4/16(0.52,I) |
| CK | >128 | 2 | 0.5 | 1 | 16 | 2/2(1.01,I) | 2/0.5(1.01,I) | 2/1(1.01,I) | 2/8(0.51,I) |
| *C. parapsilosis* | | | | | | | | | |
| ATCC22019 | >128 | 1 | 0.125 | 0.25 | 2 | 32/0.25(0.38,S) | 2/0.125(1.01,I) | 2/0.25(1.01,I) | 1/2(1.00,I) |
| CP | >128 | 2 | 0.125 | 0.5 | 16 | 2/2(1.01,I) | 2/0.125(1.01,I) | 2/0.5(1.01,I) | 4/16(1.02,I) |
| *C.tropicalis* |  |  |  |  |  |  |  |  |  |
| CT1 | >128 | 1 | 4 | 0.5 | 4 | 2/1(1.01,I) | 2/4(1.01,I) | 2/0.5(1.01,I) | 1/1(0.25,S)^d^ |
| CT2 | >128 | 0.5 | 2 | 0.5 | 4 | 2/0.5(1.01,I) | 2/2(1.01,I) | 2/0.5(1.01,I) | 2/0.5(0.13,S)^d^ |
| CT3 | >128 | 2 | 0.125 | 0.5 | 2 | 64/0.5(<0.50,S) | 2/0.125(1.01,I) | 2/0.5(1.01,I) | 1/1(0.504,I) |

^a^ The MIC is the concentration achieving 50% growth inhibition;

^b^ FICI results are shown in parentheses. S, synergy (FICI of ≤ 0.5); I, no interaction (indifference) (0.5<FICI≤4); A, antagonism (FICI of >4). For FICI calculations, the concentration of 256μg/ml were used when MICs were >128μg/ml.

^c^ Category change from resistance to susceptible dose dependent (SDD) of ITC and FLC

^d^ Category change from SDD to susceptible of ITC and FLC

^e^ Category change from resistance to susceptible of ITC and FLC

^f^ Category change from intermediate to susceptible of VRC

^g^ Category change from resistance to intermediate of VRC

Susceptible/SDD/resistant is defined as an MIC ≤2/4/≥8 mg/L of fluconazole for *C. albicans*, *C. tropicalis* and *C. parapsilosis*, respectively (1).
Susceptible/intermidiate/resistant is defined as an MIC ≤0.125/0.25-0.5/≥1 mg/L of voriconazole for *C. albicans, C. tropicalis* and *C. parapsilosis*, and an MIC ≤0.5/1/≥2 mg/L of voriconazole for *C. krusei* (1).
Susceptible/SDD/resistant is defined as an MIC ≤0.125/0.25 – 0.5/≥1 mg/L of itraconazole for *C. albicans*(1).

Category change was analyzed for those species with CLSI clinical breakpoint.

TABLE S5 Summary of drug interaction for the combination of PPIs and azoles against *Candida* spp.

| PPIs Triazoles | OME | | | PAN | | | LAN | | | RAB | | | |  |
| --- | --- | --- | --- | --- | --- | --- | --- | --- | --- | --- | --- | --- | --- | --- |
|  | S | I | A | S | I | A | S | I | A | | S | I | A | |
| ITC | 11 | 5 | 0 | 6 | 10 | 0 | 13 | 3 | 0 | | 5 | 10 | 1 | |
| POS | 11 | 5 | 0 | 7 | 9 | 0 | 11 | 5 | 0 | | 0 | 16 | 0 | |
| VRC | 2 | 14 | 0 | 0 | 16 | 0 | 1 | 15 | 0 | | 0 | 16 | 0 | |
| FLC | 8 | 8 | 0 | 8 | 8 | 0 | 5 | 11 | 0 | | 2 | 14 | 0 | |

Reference

1. Pfaller MA, Diekema DJ. 2012. Progress in antifungal susceptibility testing of Candida spp. by use of Clinical and Laboratory Standards Institute broth microdilution methods, 2010 to 2012. J Clin Microbiol 50:2846-56.

TABLE S6 MICs and FICIs results with the combinations of omeprazole and azoles against *Aspergillus* spp.

|  | MICa (mg/mL) for | | | | | | | |
| --- | --- | --- | --- | --- | --- | --- | --- | --- |
|  | Agent a alone | | | |  | Combinationb | | |
| Strains | OME | ITC | VOR | POS |  | OME/ITC | OME/VOR | OME/POS |
| *A. flavus* |  |  |  |  |  |  |  |  |
| AFLA64 | >128 | 1 | 0.5 | 1 |  | 32/0.25(0.38,S) | 2/0.5(1.01,I) | 32/0.25(0.38,S) |
| AFLA3357 | >128 | 1 | 0.5 | 1 |  | 64/0.25(0.5,S) | 2/0.5(1.01,I) | 32/0.125(0.25,S) |
| AFLA2 | >128 | 1 | 2 | 1 |  | 64/0.25(0.5,S) | 2/2(1.01,I) | 32/0.25(0.38,S) |
| AFLA3 | >128 | 1 | 0.5 | 1 |  | 32/0.25(0.38,S) | 2/2(4.01,A) | 32/0.125(0.25,S) |
| AFLA4 | >128 | 2 | 2 | 1 |  | 32/0.5(0.38,S) | 2/4(2.01,I) | 32/0.125(0.25,S) |
| AFLA5 | >128 | 1 | 0.5 | 1 |  | 32/0.25(0.38,S) | 2/0.5(1.01,I) | 32/0.125(0.25,S) |
| AFLAS25 | >128 | 1 | 0.25 | 1 |  | 32/0.125(0.25,S) | 2/0.25(1.01,I) | 32/0.125(0.25,S) |
| AFLAS32-2 | >128 | 0.5 | 2 | 1 |  | 2/0.5(1.01,I) | 2/2(1.01,I) | 16/0.125(0.19,S) |
| AFLAS32-1 | >128 | 1 | 0.5 | 1 |  | 32/0.125(0.25,S) | 2/0.5(1.01,I) | 32/0.125(0.25,S) |
| AFLAS7 | >128 | 0.5 | 0.5 | 0.5 |  | 32/0.063(0.25,S) | 2/0.5(1.01,I) | 16/0.125(0.31,S) |
| AFLA23 | >128 | 1 | 0.5 | 1 |  | 32/0.125(0.25,S) | 2/0.5(1.01,I) | 64/0.063(0.31,S) |
| AFLA109 | >128 | 1 | 2 | 1 |  | 128/0.25(0.75,I) | 2/2(1.01,I) | 16/0.25(0.31,S) |
| *A. fumigatus* | | | | | | | | |
| AF293 | >128 | 2 | 0.5 | 1 |  | 32/0.25(0.25,S) | 2/0.5(1.01,I) | 64/0.063(0.31,S) |
| AF3 | >128 | 4 | 2 | 1 |  | 32/1(0.38,S) | 2/2(1.01,I) | 32/0.25(0.38,S) |
| AF4 | >128 | 1 | 0.5 | 1 |  | 64/0.125(0.38,S) | 2/0.5(1.01,I) | 32/0.063(0.19,S) |
| AF5 | >128 | 2 | 0.5 | 1 |  | 32/0.5(0.38,S) | 2/0.5(1.01,I) | 16/0.25(0.31,S) |
| AF6 | >128 | 2 | 0.5 | 1 |  | 32/0.5(0.38,S) | 2/2(4.01,A) | 16/0.25(0.31,S) |
| AF7 | >128 | 1 | 0.5 | 1 |  | 64/0.25(0.5,S) | 2/0.5(1.01,I) | 16/0.25(0.31,S) |
| AF8 | >128 | 8 | 2 | 2 |  | 2/8(1.01,I) | 2/2(1.01,I) | 64/0.5(0.5,S) |
| AF9 | >128 | 1 | 0.25 | 0.5 |  | 64/0.25(0.5,S) | 2/0.25(1.01,I) | 16/0.063(0.19,S) |
| AF10 | >128 | 2 | 0.5 | 1 |  | 16/0.5(0.31,S) | 2/0.5(1.01,I) | 32/0.125(0.25,S) |
| AF-A | >128 | 1 | 0.5 | 0.5 |  | 64/0.125(0.38,S) | 2/0.5(1.01,I) | 16/0.063(0.19,S) |
| R1(TR34/L98H) | >128 | >32 | 8 | 2 |  | >128/>32(2,I) | 2/8(1.01,I) | 2/1(0.51,I) |
| R2(TR34/L98H) | >128 | >32 | 2 | 1 |  | >128/>32(2,I) | 2/2(1.01,I) | 64/0.5(0.75,I) |
| R3(TR34/L98H) | >128 | >32 | 8 | 2 |  | >128/>32(2,I) | 2/8(1.01,I) | 128/1(1,I) |
| R4(TR46/Y121F/T 289A) | >128 | 4 | 32 | 2 |  | 16/1(0.31,S) | 2/32(1.01,I) | 64/0.5(0.5,S) |
| *A.niger* |  |  |  |  |  |  |  |  |
| AN-1 | >128 | 1 | 1 | 1 |  | 2/1(1.01,I) | 2/1(1.01,I) | 32/0.25(0.38,S) |

^a^The MIC is the concentration achieving 100% growth inhibition;

^b^FICI results are shown in parentheses. S, synergy (FICI of ≤ 0.5); I, no interaction (indifference) (0.5<FICI≤4)；A, antagonism (FICI of >4). For FICI calculations, the next highest concentrations were used when MICs were not readable under tested concentrations.

TABLE S7 MICs and FICIs results with the combinations of pantoprazole and azoles against *Aspergillus* spp.

|  | MIC^a^ (mg/mL) for | | | | | | | |
| --- | --- | --- | --- | --- | --- | --- | --- | --- |
|  | Agent a alone | | | |  | Combination^b^ | | |
| Strains | PAN | ITC | VOR | POS |  | PAN/ITC | PAN/VOR | PAN/POS |
| *A. flavus* |  |  |  |  |  |  |  |  |
| AFLA64 | >128 | 0.5 | 0.5 | 1 |  | 16/0.25(0.56,I) | 2/0.5(1.01,I) | 64/0.125(0.38,S) |
| AFLA3357 | >128 | 0.5 | 0.5 | 0.5 |  | 64/0.125(0.5,S) | 2/0.5(1.01,I) | 64/0.125(0.5,S) |
| AFLA2 | >128 | 0.5 | 1 | 1 |  | 32/0.25(0.63,I) | 2/1(1.01,I) | 64/0.25(0.5,S) |
| AFLA3 | >128 | 1 | 0.5 | 1 |  | 32/0.25(0.38,S) | 2/0.5(1.01,I) | 64/0.25(0.5,S) |
| AFLA4 | >128 | 0.5 | 1 | 0.5 |  | 128/0.125(0.75,I) | 2/1(1.01,I) | 64/0.25(0.75,I) |
| AFLA5 | >128 | 0.5 | 0.5 | 1 |  | 128/0.125(0.75,I) | 2/0.5(1.01,I) | 64/0.25(0.5,S) |
| AFLAS25 | >128 | 0.5 | 0.5 | 1 |  | 64/0.125(0.5,S) | 2/0.5(1.01,I) | 32/0.25(0.38,S) |
| AFLAS32-2 | >128 | 0.5 | 0.5 | 0.5 |  | 64/0.125(0.5,S) | 2/0.5(1.01,I) | 64/0.125(0.5,S) |
| AFLAS32-1 | >128 | 0.5 | 0.5 | 0.5 |  | 128/0.125(0.75,I) | 2/0.5(1.01,I) | 64/0.125(0.5,S) |
| AFLAS7 | >128 | 0.5 | 0.25 | 0.5 |  | 32/0.063(0.25,S) | 2/0.25(1.01,I) | 64/0.063(0.38,S) |
| AFLA23 | >128 | 0.5 | 0.5 | 1 |  | 64/0.125(0.5,S) | 2/0.5(1.01,I) | 32/0.25(0.38,S) |
| AFLA109 | >128 | 1 | 2 | 1 |  | 128/0.25(0.75,I) | 2/2(1.01,I) | 16/0.25(0.31,S) |
| *A. fumigatus* |  |  |  |  |  |  |  |  |
| AF293 | >128 | 1 | 0.5 | 1 |  | 64/0.25(0.5,S) | 2/0.5(1.01,I) | 64/0.125(0.38,S) |
| AF3 | >128 | 1 | 1 | 1 |  | 128/0.5(1,I) | 2/1(1.01,I) | 64/0.25(0.5,S) |
| AF4 | >128 | 0.5 | 0.25 | 0.5 |  | 128/0.125(0.75,I) | 2/0.25(1.01,I) | 64/0.063(0.38,S) |
| AF5 | >128 | 1 | 0.5 | 1 |  | 32/0.25(0.38,S) | 2/0.5(1.01,I) | 64/0.125(0.38,S) |
| AF6 | >128 | 1 | 0.5 | 0.5 |  | 64/0.25(0.5,S) | 2/0.5(1.01,I) | 64/0.063(0.38,S) |
| AF7 | >128 | 1 | 0.5 | 0.5 |  | 64/0.25(0.5,S) | 2/0.5(1.01,I) | 32/0.125(0.38,S) |
| AF8 | >128 | 8 | 2 | 1 |  | 128/8(1.5,I) | 2/2(1.01,I) | 128/0.5(1,I) |
| AF9 | >128 | 1 | 0.25 | 0.5 |  | 64/0.5(0.75,I) | 2/0.25(1.01,I) | 32/0.063(0.25,S) |
| AF10 | >128 | 1 | 0.5 | 1 |  | 64/0.5(0.75,I) | 2/0.5(1.01,I) | 16/0.25(0.31,S) |
| AF-A | >128 | 0.5 | 0.25 | 0.5 |  | 64/0.25(0.75,I) | 2/0.25(1.01,I) | 32/0.125(0.38,S) |
| R1(TR34/L98H) | >128 | >32 | 8 | 2 |  | >128/>32(2,I) | 2/8(1.01,I) | 2/1(0.508,I) |
| R2(TR34/L98H) | >128 | >32 | 2 | 1 |  | >128/>32(2,I) | 2/2(1.01,I) | 6 4/0.5(0.75,I) |
| R3(TR34/L98H) | >128 | >32 | 8 | 2 |  | >128/>32(2,I) | 2/8(1.01,I) | 2/2(1.01,I) |
| R4(TR46/Y121F/T 289A) | >128 | 4 | 32 | 2 |  | 64/1(0.5,S) | 2/32(1.01,I) | 64/0.5(0.5,S) |
| *A.niger* |  |  |  |  |  |  |  |  |
| AN-1 | >128 | 1 | 1 | 1 |  | 128/0.5(1,I) | 2/1(1.01,I) | 64/0.25(0.5,S) |

^a^The MIC is the concentration achieving 100% growth inhibition;

^b^FICI results are shown in parentheses. S, synergy (FICI of ≤ 0.5); I, no interaction (indifference) (0.5<FICI≤4); A, antagonism (FICI of >4). For FICI calculations, the next highest concentrations were used when MICs were not readable under tested concentrations.

TABLE S8 MICs and FICIs results with the combinations of lansoprazole and azoles against *Aspergillus* spp.

|  | MIC^a^ (mg/mL) for | | | | | | | | | | | | | | | | | |
| --- | --- | --- | --- | --- | --- | --- | --- | --- | --- | --- | --- | --- | --- | --- | --- | --- | --- | --- |
|  | Agent a alone | | | | | | |  | | | | Combination^b^ | | | | | | |
| Strains | LAN | | ITC | | VOR | | POS | | |  | | | LAN/ITC | | LAN/VOR | | LAN/POS | |
| *A. flavus* |  | |  | |  | |  | | |  | | |  | |  | |  | |
| AFLA64 | >128 | | 0.5 | | 1 | | 1 | | |  | | | 32/0.125(0.38,S) | | 2/1(1.01,I) | | 8/0.25(0.281,S) | |
| AFLA3357 | >128 | | 0.5 | | 0.5 | | 0.5 | | |  | | | 32/0.063(0.25,S) | | 2/0.5(1.01,I) | | 16/0.125(0.31,S) | |
| AFLA2 | >128 | | 0.5 | | 2 | | 1 | | |  | | | 32/0.063(0.25,S) | | 2/2(1.01,I) | | 16/0.25(0.31,S) | |
| AFLA3 | >128 | | 1 | | 0.5 | | 0.5 | | |  | | | 32/0.063(0.19,S) | | 2/0.5(1.01,I) | | 32/0.125(0.38,S) | |
| AFLA4 | >128 | | 0.5 | | 2 | | 1 | | |  | | | 32/0.063(0.25,S) | | 2/4(2.01,I) | | 16/0.25(0.31,S) | |
| AFLA5 | >128 | | 0.5 | | 0.5 | | 1 | | |  | | | 32/0.063(0.25,S) | | 2/0.5(1.01,I) | | 16/0.25(0.31,S) | |
| AFLAS25 | >128 | | 0.5 | | 0.5 | | 1 | | |  | | | 32/0.063(0.25,S) | | 2/0.5(1.01,I) | | 16/0.25(0.31,S) | |
| AFLAS32-2 | >128 | | 0.25 | | 2 | | 0.5 | | |  | | | 32/0.063(0.38,S) | | 2/2(1.01,I) | | 16/0.063(0.19,S) | |
| AFLAS32-1 | >128 | | 0.5 | | 0.5 | | 0.5 | | |  | | | 16/0.063(0.19,S) | | 2/0.5(1.01,I) | | 16/0.063(0.19,S) | |
| AFLAS7 | >128 | | 0.5 | | 0.5 | | 1 | | |  | | | 32/0.125(0.38,S) | | 2/0.5(1.01,I) | | 8/0.125(0.156,S) | |
| AFLA23 | >128 | | 0.5 | | 0.5 | | 1 | | |  | | | 32/0.063(0.25,S) | | 2/0.5((1.01,I)) | | 32/0.063(0.19,S) | |
| AFLA109 | >128 | | 1 | | 2 | | 1 | | |  | | | 128/0.25(0.75,I) | | 2/2((1.01,I)) | | 32/0.25(0.38,S) | |
| *A. fumigatus* |  | |  | |  | |  | | |  | | |  | |  | |  | |
| AF293 | >128 | | 1 | | 0.5 | | 1 | | |  | | | 32/0.125(0.25,S) | | 2/0.5(1.01,I) | | 32/0.0625(0.19,S) | |
| AF3 | >128 | | 1 | | 2 | | 1 | | |  | | | 64/0.25(0.5,S) | | 2/2(1.01,I) | | 16/0.25(0.31,S) | |
| AF4 | >128 | | 0.5 | | 0.5 | | 0.5 | | |  | | | 32/0.125(0.38,S) | | 2/0.5(1.01,I) | | 16/0.063(0.19,S) | |
| AF5 | >128 | | 1 | | 0.5 | | 1 | | |  | | | 32/0.125(0.25,S) | | 2/0.5(1.01,I) | | 16/0.063(0.13,S) | |
| AF6 | >128 | | 1 | | 0.5 | | 1 | | |  | | | 32/0.125(0.25,S) | | 2/0.5(1.01,I) | | 16/0.063(0.13,S) | |
| AF7 | >128 | | 0.5 | | 0.5 | | 1 | | |  | | | 32/0.125(0.38,S) | | 2/0.5(1.01,I) | | 16/0.063(0.13,S) | |
| AF8 | >128 | | 8 | | 2 | | 1 | | |  | | | 2/8(1.01,I) | | 2/2(1.01,I) | | 16/0.5(0.56,I) | |
| AF9 | >128 | | 1 | | 0.25 | | 0.5 | | |  | | | 32/0.25(0.38,S) | | 2/0.25((1.01,I)) | | 8/0.125(0.28,S) | |
| AF10 | >128 | | 1 | | 0.5 | | 1 | | |  | | | 16/0.25(0.31,S) | | 2/0.5((1.01,I)) | | 16/0.063(0.13,S) | |
| AF-A | >128 | | 1 | | 0.5 | | 0.5 | | |  | | | 32/0.125(0.25,S) | | 2/0.5((1.01,I)) | | 16/0.063(0.19,S) | |
| R1(TR34/L98H) | >128 | >32 | | 8 | | 2 | | |  | | >128/>32(2,I) | | | 2/8((1.01,I)) | | 32/0.5(0.38,S) | |  |
| R2(TR34/L98H) | >128 | | >32 | | 2 | | 1 | | |  | | | >128/>32(2,I) | | 2/2((1.01,I)) | | 16/0.5(0.56,I) | |
| R3(TR34/L98H) | >128 | | >32 | | 8 | | 2 | | |  | | | >128/>32(2,I) | | 2/8((1.01,I)) | | 32/1(0.63,I) | |
| R4(TR46/Y121F/T 289A) | >128 | | 4 | | 32 | | 2 | | |  | | | 32/0.5(0.25,S) | | 2/32((1.01,I)) | | 16/0.5(0.31,S) | |
| *A. niger* |  | |  | |  | |  | | |  | | |  | |  | |  | |
| AN-1 | >128 | | 1 | | 1 | | 1 | | |  | | | 64/0.25(0.5,S) | | 2/1((1.01,I)) | | 32/0.125(0.25,S) | |

^a^The MIC is the concentration achieving 100% growth inhibition;

^b^FICI results are shown in parentheses. S, synergy (FICI of ≤ 0.5); I, no interaction (indifference) (0.5<FICI≤4); A, antagonism (FICI of >4). For FICI calculations, the next highest concentrations were used when MICs were not readable under tested concentrations.

TABLE S9 MICs and FICIs results with the combinations of Rabeprazole and azoles against *Aspergillus* spp.

|  | MIC^a^ (mg/mL) for | | | | | | | | | |  |
| --- | --- | --- | --- | --- | --- | --- | --- | --- | --- | --- | --- |
|  | Agent a alone | | | |  | | Combination^b^ | | | |  |
| Strains | RAB | ITC | VOR | POS | |  | | RAB/ITC | RAB/VOR | RAB/POS | |
| *A. flavus* |  |  |  |  | |  | |  |  |  | |
| AFLA64 | >128 | 1 | 0.5 | 1 | |  | | 2/1(1.01,I) | 2/0.5(1.01,I) | 2/1(1.01,I) | |
| AFLA3357 | >128 | 2 | 0.5 | 1 | |  | | 2/2(1.01,I) | 2/0.5(1.01,I) | 32/0.25(0.38,S) | |
| AFLA2 | >128 | 2 | 1 | 1 | |  | | 2/2(1.01,I) | 2/1((1.01,I)) | 32/0.125(0.25,S) | |
| AFLA3 | >128 | 2 | 0.25 | 1 | |  | | 64/0.5(0.5,S) | 2/0.25(1.01,I) | 32/0.063(0.19,S) | |
| AFLA4 | >128 | 4 | 0.5 | 1 | |  | | 64/1(0.5,S) | 32/0.25(0.63,I) | 64/0.063(0.31,S) | |
| AFLA5 | >128 | 2 | 0.25 | 1 | |  | | 32/0.5(0.38,S) | 2/0.25(1.01,I) | 32/0.063(0.19,S) | |
| AFLAS25 | >128 | 2 | 0.5 | 1 | |  | | 2/2(1.01,I) | 2/0.5(1.01,I) | 64/0.125(0.38,S) | |
| AFLAS32-2 | >128 | 1 | 0.5 | 1 | |  | | 2/1(1.01,I) | 2/0.5(1.01,I) | 32/0.125(0.25,S) | |
| AFLAS32-1 | >128 | 1 | 0.5 | 0.5 | |  | | 2/1(1.01,I) | 2/0.5(1.01,I) | 64/0.125(0.5,S) | |
| AFLAS7 | >128 | 1 | 0.5 | 0.5 | |  | | 2/1(1.01,I) | 2/0.5(1.01,I) | 32/0.125(0.38,S) | |
| AFLA23 | >128 | 0.5 | 1 | 1 | |  | | 2/0.5(1.01,I) | 2/1(1.01,I) | 64/0.125(0.38,S) | |
| AFLA109 | >128 | 1 | 2 | 1 | |  | | 128/0.25(0.75,I) | 2/2(1.01,I) | 64/0.25(0.5,S) | |
| *A. fumigatus* |  |  |  |  | |  | |  |  |  | |
| AF293 | >128 | 2 | 0.5 | 1 | |  | | 32/1(0.63,I) | 2/0.5(1.01,I) | 32/0.25(0.38,S) | |
| AF3 | >128 | 2 | 0.5 | 1 | |  | | 64/0.5(0.5,S) | 2/0.5(1.01,I) | 16/0.25(0.31,S) | |
| AF4 | >128 | 1 | 0.5 | 1 | |  | | 64/0.25(0.5,S) | 2/0.5(1.01,I) | 16/0.25(0.31,S) | |
| AF5 | >128 | 8 | 2 | 2 | |  | | 2/8(1.01,I) | 2/2(1.01,I) | 64/0.5(0.5,S) | |
| AF6 | >128 | 2 | 1 | 1 | |  | | 2/2(1.01,I) | 2/1(1.01,I) | 64/0.063(0.31,S) | |
| AF7 | >128 | 2 | 0.5 | 1 | |  | | 64/0.5(0.5,S) | 2/0.5(1.01,I) | 32/0.125(0.25,S) | |
| AF8 | >128 | 2 | 1 | 1 | |  | | 2/2(1.01,I) | 2/1(1.01,I) | 32/0.25(0.38,S) | |
| AF9 | >128 | 1 | 0.25 | 0.5 | |  | | 16/0.5(0.56,I) | 2/0.25(1.01,I) | 64/0.125(0.5,S) | |
| AF10 | >128 | 2 | 0.5 | 1 | |  | | 64/0.5(0.5,S) | 2/0.5(1.01,I) | 32/0.25(0.38,S) | |
| AF-A | >128 | 2 | 0.25 | 1 | |  | | 32/0.5(0.38,S) | 2/0.25(1.01,I) | 32/0.063(0.19,S) | |
| R1(TR34/L98H) | >128 | >32 | 8 | 2 | |  | | >128/>32(2,I) | 2/8(1.01,I) | 2/1(0.508,I) | |
| R2(TR34/L98H) | >128 | >32 | 2 | 1 | |  | | >128/>32(2,I) | 2/2(1.01,I) | 32/0.25(0.38,S) | |
| R3(TR34/L98H) | >128 | >32 | 8 | 2 | |  | | >128/>32(2,I) | 2/8(1.01,I) | 2/2(1.01,I) | |
| R4(TR46/Y121F/T 289A) | >128 | 4 | 32 | 2 | |  | | 64/0.5(0.38,S) | 2/32(1.01,I) | 64/0.5(0.5,S) | |
| *A.niger* |  |  |  |  | |  | |  |  |  | |
| AN-1 | >128 | 1 | 1 | 1 | |  | | 2/1(1.01,I) | 2/1(1.01,I) | 64/0.25(0.5,S) | |

^a^The MIC is the concentration achieving 100% growth inhibition;

^b^FICI results are shown in parentheses. S, synergy (FICI of ≤ 0.5); I, no interaction (indifference) (0.5<FICI≤4) ; A, antagonism (FICI of >4). For FICI calculations, the next highest concentrations were used when MICs were not readable under tested concentrations.

TABLE S10 Summary of drug interaction for the combination of PPIs and azoles against *Aspergillus* spp.

| Drug Combination | | *A. flavus*(n=12) | | | *A. fumigatus*(n=14) | | | *A. nigra*(n=1) | | | | Total(n=27) | | | |
| --- | --- | --- | --- | --- | --- | --- | --- | --- | --- | --- | --- | --- | --- | --- | --- |
|  |  | S | I | A | S | I | A | S | I | A | S | | I | A |  |
| ITC | OME | 10 | 2 | 0 | 10 | 4 | 0 | 0 | 1 | 0 | 20 | | 7 | 0 |  |
|  | PAN | 6 | 6 | 0 | 5 | 9 | 0 | 0 | 1 | 0 | 11 | | 16 | 0 |  |
|  | LAN | 11 | 1 | 0 | 10 | 4 | 0 | 1 | 0 | 0 | 22 | | 5 | 0 |  |
|  | RAB | 3 | 9 | 0 | 6 | 8 | 0 | 0 | 1 | 0 | 9 | | 18 | 0 |  |
| POS | OME | 12 | 0 | 0 | 11 | 3 | 0 | 1 | 0 | 0 | 24 | | 3 | 0 |  |
|  | PAN | 11 | 1 | 0 | 10 | 4 | 0 | 1 | 0 | 0 | 22 | | 5 | 0 |  |
|  | LAN | 12 | 0 | 0 | 11 | 3 | 0 | 1 | 0 | 0 | 24 | | 3 | 0 |  |
|  | RAB | 11 | 1 | 0 | 12 | 2 | 0 | 1 | 0 | 0 | 24 | | 3 | 0 |  |
| VRC | OME | 0 | 11 | 1 | 0 | 13 | 1 | 0 | 1 | 0 | 0 | | 25 | 2 |  |
|  | PAN | 0 | 12 | 0 | 0 | 14 | 0 | 0 | 1 | 0 | 0 | | 27 | 0 |  |
|  | LAN | 0 | 12 | 0 | 0 | 14 | 0 | 0 | 1 | 0 | 0 | | 27 | 0 |  |
|  | RAB | 0 | 12 | 0 | 0 | 14 | 0 | 0 | 1 | 0 | 0 | | 27 | 0 |  |

TABLE S11 MICs and FICIs results with the combinations of omeprazole and azoles against dematiaceous fungi

|  | MICa (mg/mL) for | | | | | | |
| --- | --- | --- | --- | --- | --- | --- | --- |
|  | Agent alone | | | | Combinationb | | |
| Strains | OME | ITC | VOR | POS | OME/ITC | OME/VOR | OME/POS |
| *Exophiala dermatitidis* | | | | | | | |
| BMU00028 | >128 | 1 | 0.25 | 0.5 | 32/0.25(0.38,S) | 2/0.25(1.01,I) | 32/0.0625(0.25,S) |
| BMU00029 | >128 | 2 | 1 | 1 | 64/0.25(0.38,S) | 128/0.5(1.00,I) | 64/0.25(＜0.50,S) |
| BMU00030 | >128 | 0.5 | 0.125 | 0.5 | 16/0.0625(0.19,S) | 2/0.125(1.01,I) | 16/0.125(0.31,S) |
| BMU00031 | >128 | 1 | 0.5 | 1 | 64/0.125(0.38,S) | 2/0.5(1.01,I) | 32/0.0625(0.19,S) |
| BMU00034 | >128 | 2 | 0.125 | 1 | 16/0.125(0.13,S) | 2/0.5(4.01,A) | 32/0.25(0.38,S) |
| BMU00035 | >128 | 0.5 | 0.125 | 0.5 | 32/0.125(0.38,S) | 2/0.125(1.01,I) | 16/0.0625(0.19,S) |
| BMU00036 | >128 | 1 | 0.125 | 0.5 | 16/0.25(0.31,S) | 2/0.125(1.01,I) | 64/0.125(＜0.50,S) |
| BMU00037 | >128 | 1 | 0.25 | 0.5 | 32/0.125(0.25,S) | 2/0.25(1.01,I) | 16/0.0625(0.19,S) |
| BMU00038 | >128 | 1 | 0.125 | 0.5 | 32/0.125(0.25,S) | 2/0.25(2.01,I) | 16/0.0625(0.19,S) |
| BMU00039 | >128 | 1 | 0.25 | 1 | 32/0.125(0.25,S) | 2/0.125(0.51,I) | 16/0.03125(0.09,S) |
| BMU00040 | >128 | 0.5 | 0.25 | 0.25 | 16/0.125(0.31,S) | 2/0.0625(0.26,S) | 8/0.0625(0.28,S) |
| BMU00041 | >128 | 0.5 | 0.125 | 0.5 | 16/0.125(0.31,S) | 2/0.125(1.01,I) | 8/0.0625(0.16,S) |
| 109140 | >128 | 0.5 | 0.125 | 0.5 | 64/0.125(＜0.5,S) | 2/0.25(2.01,I) | 32/0.0625(0.25,S) |
| 109142 | >128 | 0.5 | 0.125 | 0.5 | 16/0.25(0.56,I) | 2/0.125(1.01,I) | 16/0.125(0.31,S) |
| 109144 | >128 | 1 | 2 | 0.5 | 16/0.125(0.19,S) | 2/0.125(0.07,S) | 32/0.03125(0.19,S) |
| 109145 | >128 | 1 | 0.25 | 0.5 | 16/0.125(0.19,S) | 2/0.25(1.01,I) | 16/0.125(0.31,S) |
| 109149 | >128 | 0.5 | 0.125 | 0.5 | 16/0.25(0.56,I) | 2/0.125(1.01,I) | 16/0.125(0.31,S) |
| 109152 | >128 | 0.125 | 0.25 | 0.5 | 16/0.0625(0.56,I) | 2/0.25(1.01,I) | 8/0.0625(0.16,S) |
| *Phialophora macrospora* | | | | | | | |
| CBS00106 | >128 | 1 | 0.125 | 0.5 | 16/0.125(0.19,S) | 2/0.125(1.01,I) | 32/0.0625(0.25,S) |
| *Phialophora americana* | | | | | | | |
| CBS 00107 | >128 | 2 | 0.25 | 0.5 | 16/0.5(0.31,S) | 2/0.25(1.01,I) | 16/0.125(0.31,S) |
| CBS 00109 | 64 | 1 | 1 | 1 | 16/0.125(0.19,S) | 2/0.5(0.51,I) | 32/0.125(0.25,S) |
| *Fonsecaea monophora* | | | | | | | |
| BMU07631 | >128 | 0.5 | 0.25 | 0.5 | 32/0.0625(0.25,S) | 2/0.25(1.01,I) | 16/0.125(0.31,S) |
| BMU07632 | >128 | 0.5 | 0.0625 | 0.5 | 32/0.03125(0.19,S) | 2/0.0625(1.01,I) | 16/0.25(0.56,I) |
| BMU07633 | 64 | 1 | 0.0625 | 1 | 16/0.25(0.50,S) | 2/0.0625(1.01,I) | 16/0.0625(0.13,S) |

^a^The MIC is the concentration achieving 100% growth inhibition;

^b^FICI results are shown in parentheses. S, synergy (FICI of ≤ 0.5); I, no interaction (indifference) (0.5<FICI≤4); A, antagonism (FICI of >4). For FICI calculations, the next highest concentrations were used when MICs were not readable under tested concentrations.

TABLE S12 MICs and FICIs results with the combinations of pantoprazole and azoles against dematiaceous fungi

|  | MICa (mg/mL) for | | | | | | |
| --- | --- | --- | --- | --- | --- | --- | --- |
|  | Agent alone | | | | Combinationb | | |
| Strains | PAN | ITC | VOR | POS | PAN/ITC | PAN/VOR | PAN/POS |
| *E. dermatitidis* | | | | | | | |
| BMU00028 | >128 | 1 | 0.25 | 0.5 | 32/1(1.13,I) | 2/0.25(1.01,I) | 32/0.125(0.38,S) |
| BMU00029 | >128 | 2 | 1 | 1 | 2/2(1.01,I) | 2/1(1.01,I) | 64/0.25(0.50,S) |
| BMU00030 | >128 | 0.5 | 0.125 | 0.5 | 32/0.125(0.38,S) | 2/0.125(1.01,I) | 16/0.125(0.31,S) |
| BMU00031 | >128 | 1 | 0.5 | 1 | 32/0.5(0.63,I) | 2/0.5(1.01,I) | 64/0.125(0.38,S) |
| BMU00034 | >128 | 2 | 0.125 | 1 | 16/1(0.56,I) | 16/0.25(2.06,I) | 64/0.25(0.50,S) |
| BMU00035 | >128 | 0.5 | 0.125 | 0.5 | 64/0.125(＜0.50,S) | 2/0.25(2.01,I) | 32/0.0625(0.25,S) |
| BMU00036 | >128 | 1 | 0.125 | 0.5 | 32/0.25(0.38,S) | 2/0.125(1.01,I) | 16/0.125(0.31,S) |
| BMU00037 | >128 | 1 | 0.25 | 0.5 | 64/0.125(0.38,S) | 2/0.25(1.01,I) | 32/0.0625(0.25,S) |
| BMU00038 | >128 | 1 | 0.125 | 0.5 | 32/0.25(0.38,S) | 2/0.125(1.01,I) | 32/0.125(0.38,S) |
| BMU00039 | >128 | 1 | 0.25 | 1 | 64/0.25(＜0.50,S) | 2/0.25(1.01,I) | 32/0.125(0.25,S) |
| BMU00040 | >128 | 0.5 | 0.25 | 0.25 | 64/0.0625(0.38,S) | 2/0.125(0.51,I) | 32/0.125(0.63,I) |
| BMU00041 | >128 | 0.5 | 0.125 | 0.5 | 64/0.125(＜0.50,S) | 2/0.125(1.01,I) | 16/0.125(0.31,S) |
| 109140 | >128 | 0.5 | 0.125 | 0.5 | 2/0.5(1.01,I) | 2/0.25(2.01,I) | 64/0.0625(0.38,S) |
| 109142 | >128 | 0.5 | 0.125 | 0.5 | 32/0.25(0.63,I) | 2/0.0625(0.51,I) | 16/0.125(0.31,S) |
| 109144 | >128 | 1 | 2 | 0.5 | 64/0.125(0.38,S) | 2/0.125(0.07,S) | 16/0.125(0.31,S) |
| 109145 | >128 | 1 | 0.25 | 0.5 | 32/0.25(0.38,S) | 2/0.25(1,01,I) | 32/0.125(0.38,S) |
| 109149 | >128 | 0.5 | 0.125 | 0.5 | 32/0.25(0.63,I) | 2/0.25(2.01,I) | 32/0.125(0.38,S) |
| 109152 | >128 | 0.125 | 0.25 | 0.5 | 64/0.03125(＜0.50,S) | 2/0.25(1.01,I) | 16/0.03125(0.13,S) |
| *P. macrospora* | | | | | | | |
| CBS00106 | >128 | 1 | 0.125 | 0.5 | 2/0.5(0.51,I) | 2/0.125(1.01,I) | 32/0.0625(0.25,S) |
| *P. americana,* | | | | | | | |
| CBS 00107 | >128 | 2 | 0.25 | 0.5 | 64/0.5(＜0.50,S) | >128/0.25(2.00,I) | 32/0.125(0.38,S) |
| CBS 00109 | >128 | 1 | 1 | 1 | 32/0.5(0.63,I) | 2/0.5(0.51, I) | 64/0.125(0.38,S) |
| *F. monophora* | | | | | | | |
| BMU07631 | >128 | 0.5 | 0.25 | 0.5 | 64/0.125(＜0.50,S) | 2/0.25(1.01,I) | 32/0.125(0.38,S) |
| BMU07632 | >128 | 0.5 | 0.0625 | 0.5 | 64/0.125(＜0.50,S) | 2/0.0625(1.01,I) | 32/0.0625(0.25,S) |
| BMU07633 | 64 | 1 | 0.0625 | 1 | 32/0.25(0.38,S) | 2/0.125(2.01,I) | 16/0.25(0.31,S) |

^a^The MIC is the concentration achieving 100% growth inhibition;

^b^FICI results are shown in parentheses. S, synergy (FICI of ≤ 0.5); I, no interaction (indifference) (0.5<FICI≤4); A, antagonism (FICI of >4). For FICI calculations, the next highest concentrations were used when MICs were not readable under tested concentrations.

TABLE S13 MICs and FICIs results with the combinations of lansoprazole and azoles against dematiaceous fungi

|  | MICa (mg/mL) for | | | | | | |
| --- | --- | --- | --- | --- | --- | --- | --- |
|  | Agent a alone | | | | Combinationb | | |
| Strains | LAN | ITC | VOR | POS | LAN/ITC | LAN/VOR | LAN/POS |
| *E. dermatitidis* | | | | | | | |
| BMU00028 | >128 | 1 | 0.25 | 0.5 | 32/0.125(0.25,S) | 2/0.25(1.01,I) | 16/0.0625(0.19,S) |
| BMU00029 | >128 | 2 | 1 | 1 | 32/0.5(0.38,S) | >128/0.25(1.25, I) | 32/0.0625(0.19, S) |
| BMU00030 | >128 | 0.5 | 0.125 | 0.5 | 16/0.0625(0.19,S) | 2/0.125(1.01,I) | 8/0.125(0.28,S) |
| BMU00031 | >128 | 1 | 0.5 | 1 | 16/0.25(0.31,S) | 2/0.5(1.01,I) | 16/0.125(0.19,S) |
| BMU00034 | >128 | 2 | 0.125 | 1 | 32/0.5(0.38,S) | 2/0.25(2.01,I) | 8/0.5(0.53,I) |
| BMU00035 | >128 | 0.5 | 0.125 | 0.5 | 16/0.125(0.31,S) | 2/0.125(1.01,I) | 16/0.03125(0.13,S) |
| BMU00036 | >128 | 1 | 0.125 | 0.5 | 16/0.125(0.19,S) | 2/0.125(1.01,I) | 16/0.0625(0.19,S) |
| BMU00037 | >128 | 1 | 0.25 | 0.5 | 32/0.125(0.25,S) | 2/0.25(1.01,I) | 16/0.125(0.31,S) |
| BMU00038 | >128 | 1 | 0.125 | 0.5 | 32/0.125(0.25,S) | 2/0.5(4.01,A) | 16/0.125(0.31,S) |
| BMU00039 | >128 | 1 | 0.25 | 1 | 32/0.125(0.25,S) | 2/0.25(1.01,I) | 16/0.0625(0.13,S) |
| BMU00040 | >128 | 0.5 | 0.25 | 0.25 | 32/0.0625(0.25,S) | 2/0.125(0.51,I) | 16/0.03125(0.19,S) |
| BMU00041 | >128 | 0.5 | 0.125 | 0.5 | 16/0.125(0.31,S) | 2/0.125(1.01,I) | 16/0.03125(0.13,S) |
| 109140 | >128 | 0.5 | 0.125 | 0.5 | 64/0.125(0.50,S) | 2/0.25(2.01,I) | 32/0.125(0.38,S) |
| 109142 | >128 | 0.5 | 0.125 | 0.5 | 32/0.125(0.38,S) | 2/0.125(1.01,I) | 8/0.0625(0.16,S) |
| 109144 | >128 | 1 | 2 | 0.5 | 16/0.0625(0.13,S) | 2/0.125(0.07,S) | 8/0.125(0.28,S) |
| 109145 | >128 | 1 | 0.25 | 0.5 | 32/0.125(0.25,S) | 2/0.25(1.01,I) | 16/0.125(0.31,S) |
| 109149 | >128 | 0.5 | 0.125 | 0.5 | 32/0.125(0.38,S) | 2/0.125(1.01,I) | 16/0.0625(0.19,S) |
| 109152 | >128 | 0.125 | 0.25 | 0.5 | 16/0.03125(0.31,S) | 2/0.25(1.01,I) | 8/0.03125(0.09,S) |
| *P. macrospora* | | | | | | | |
| CBS00106 | >128 | 1 | 0.125 | 0.5 | 16/0.5(0.56,I) | 2/0.25(2.01,I) | 16/0.125(0.31,S) |
| *P. americana,* | | | | | | | |
| CBS 00107 | >128 | 2 | 0.25 | 0.5 | 32/0.125(0.19,S) | 2/0.25(1.01,I) | 8/0.125(0.28,S) |
| CBS 00109 | 64 | 1 | 1 | 1 | 16/0.031(0.09,S) | 2/1(1.01,I) | 16/0.0.125(0.19,S) |
| *F. monophora* | | | | | | | |
| BMU07631 | >128 | 0.5 | 0.25 | 0.5 | 16/0.0625(0.19,S) | 2/0.125(0.51,I) | 16/0.0625(0.19,S) |
| BMU07632 | >128 | 0.5 | 0.0625 | 0.5 | 16/0.03125(0.13,S) | 2/0.125(2.01,I) | 16/0.03125(0.13,S) |
| BMU07633 | 64 | 1 | 0.0625 | 1 | 16/0.063(0.13,S) | 2/0.125(2.01,I) | 16/0.03125(0.09,S) |

^a^The MIC is the concentration achieving 100% growth inhibition;

^b^FICI results are shown in parentheses. S, synergy (FICI of ≤ 0.5); I, no interaction (indifference) (0.5<FICI≤4); A, antagonism (FICI of >4). For FICI calculations, the next highest concentrations were used when MICs were not readable under tested concentrations.

TABLE S14 MICs and FICIs results with the combinations of rabeprazole and azoles against dematiaceous fungi

|  | MICa (mg/mL) for | | | | | | | | | | | |
| --- | --- | --- | --- | --- | --- | --- | --- | --- | --- | --- | --- | --- |
|  | Agent alone | | | | | Combinationb | | | | | | |
| Strains | RAB | ITC | VOR | POS | RAB/ITC | | | RAB/VOR | | RAB/POS | |  |
| *E. dermatitidis* | | | | | | | | | | | | |
| BMU00028 | >128 | 1 | 0.25 | 0.5 | 2/1(1.01,I) | | 2/0.25(1.01,I) | | 64/0.0625(0.38,S) | |  |  |
| BMU00029 | >128 | 2 | 1 | 1 | 32/1(0.63,I) | | 8/0.5(0.53,I) | | 32/0.5(0.63,I) | |  |  |
| BMU00030 | >128 | 0.5 | 0.125 | 0.5 | 2/0.5(1.01,I) | | 2/0.25(2.01,I) | | 32/0.3125(0.19,S) | |  |  |
| BMU00031 | >128 | 1 | 0.5 | 1 | 2/1(1.01,I) | | 32/0.125(0.38,S) | | 64/0.125(0.38,S) | |  |  |
| BMU00034 | >128 | 2 | 0.125 | 1 | >128/1(1.5,I) | | 2/0.5(4.01,A) | | >128/0.25(1.25,I) | |  |  |
| BMU00035 | >128 | 0.5 | 0.125 | 0.5 | 2/0.5(1.01,I) | | 2/0.125(1.01,I) | | 64/0.03125(0.31,S) | |  |  |
| BMU00036 | >128 | 1 | 0.125 | 0.5 | 2/0.5(0.51,I) | | 2/0.125(1.01,I) | | 32/0.125(0.38,S) | |  |  |
| BMU00037 | >128 | 1 | 0.25 | 0.5 | 2/1(1.01,I) | | 2/0.25(1.01,I) | | 64/0.125(＜0.5,S) | |  |  |
| BMU00038 | >128 | 1 | 0.125 | 0.5 | 2/1(1.01,I) | | 2/0.125(1.01,I) | | 64/0.125(＜0.5,S) | |  |  |
| BMU00039 | >128 | 1 | 0.25 | 1 | 2/1(1.01,I) | | 2/0.25(1.01,I) | | 32/0.25(0.38,S) | |  |  |
| BMU00040 | >128 | 0.5 | 0.25 | 0.25 | 2/0.5(1.01,I) | | 2/0.125(0.51,I) | | 16/0.0625(0.31, S) | |  |  |
| BMU00041 | >128 | 0.5 | 0.125 | 0.5 | 2/1(2.01,I) | | 2/0.5(4.01,A) | | 64/0.03125(0.31,S) | |  |  |
| 109140 | >128 | 0.5 | 0.125 | 0.25 | 2/0.5(1.01,I) | | 2/0.125(1.01,I) | | 32/0.25(1.13, I) | |  |  |
| 109142 | >128 | 0.5 | 0.125 | 0.5 | 2/0.5(1.01,I) | | 2/0.125(1.01,I) | | 32/0.25(0.63,I) | |  |  |
| 109144 | >128 | 1 | 2 | 0.5 | 2/1(1.01,I) | | 2/0.125(0.07,S) | | 32/0.125(0.38,S) | |  |  |
| 109145 | >128 | 1 | 0.25 | 0.5 | 2/1(1.01,I) | | 2/0.25(1.01,I) | | 64/0.03125(0.31,S) | |  |  |
| 109149 | >128 | 0.5 | 0.125 | 0.5 | 2/1(2.01,I) | | 2/0.25(2.01,I) | | 32/0.25(0.63,I) | |  |  |
| 109152 | >128 | 0.125 | 0.25 | 0.5 | 64/0.125(1.25,I) | | 2/0.25(1.01,I) | | 16/0.125(0.31,S) | |  |  |
| *P. macrospora* | | | | | | | | | | | | |
| CBS00106 | >128 | 1 | 0.125 | 0.5 | 16/0.5(0.56,I) | | | 2/0.25(2.01,I) | | 32/0.125(0.38,S) | |  |
| *P. americana,* | | | | | | | | | | | | |
| CBS 00107 | >128 | 2 | 0.25 | 0.5 | 32/0.5(0.38,S) | | | 2/0.5(2.01,I) | | 32/0.125(0.38,S) | |  |
| CBS 00109 | >128 | 1 | 1 | 1 | 64/0.5(0.75,I) | | | 2/1(1.01,I) | | 32/0.25(0.38, S) | |  |
| *F. monophora* | | | | | | | | | | | | |
| BMU07631 | >128 | 0.5 | 0.25 | 0.5 | 64/0.25(0.75,I) | | | 2/0.25(1.01,I) | | 32/0.125(0.38,S) | |  |
| BMU07632 | >128 | 0.5 | 0.125 | 0.5 | 2/0.5(1.01,I) | | | 2/0.125(1.01,I) | | 32/0.0625(0.25,S) | |  |
| BMU07633 | >128 | 1 | 0.0625 | 1 | 64/0.25(＜0.50,S) | | | 2/0.063(1.02,I) | | 4/0.25(0.27,S) | |  |

^a^The MIC is the concentration achieving 100% growth inhibition;

^b^FICI results are shown in parentheses. S, synergy (FICI of ≤ 0.5); I, no interaction (indifference) (0.5<FICI≤4); A, antagonism (FICI of >4). For FICI calculations, the next highest concentrations were used when MICs were not readable under tested concentrations.

| PPIs Triazoles | OME | | | PAN | | | LAN | | | RAB | | | |  |
| --- | --- | --- | --- | --- | --- | --- | --- | --- | --- | --- | --- | --- | --- | --- |
|  | S | I | A | S | I | A | S | I | A | | S | I | A | |
| ITC | 21 | 3 | 0 | 15 | 9 | 0 | 23 | 1 | 0 | | 2 | 22 | 0 | |
| POS | 23 | 1 | 0 | 24 | 0 | 0 | 23 | 1 | 0 | | 19 | 5 | 0 | |
| VRC | 2 | 21 | 1 | 1 | 23 | 0 | 1 | 22 | 1 | | 2 | 20 | 2 | |

TABLE S15 Summary of drug interaction for the combination of PPIs and azoles against dematiaceous fungi
